# Predicting microbial growth dynamics in response to nutrient availability

**DOI:** 10.1371/journal.pcbi.1008817

**Published:** 2021-03-18

**Authors:** Olga A. Nev, Richard J. Lindsay, Alys Jepson, Lisa Butt, Robert E. Beardmore, Ivana Gudelj

**Affiliations:** Biosciences and Living Systems Institute, University of Exeter, Exeter, United Kingdom; Max-Planck-Institute for Evolutionary Biology, GERMANY

## Abstract

Developing mathematical models to accurately predict microbial growth dynamics remains a key challenge in ecology, evolution, biotechnology, and public health. To reproduce and grow, microbes need to take up essential nutrients from the environment, and mathematical models classically assume that the nutrient uptake rate is a saturating function of the nutrient concentration. In nature, microbes experience different levels of nutrient availability at all environmental scales, yet parameters shaping the nutrient uptake function are commonly estimated for a single initial nutrient concentration. This hampers the models from accurately capturing microbial dynamics when the environmental conditions change. To address this problem, we conduct growth experiments for a range of micro-organisms, including human fungal pathogens, baker’s yeast, and common coliform bacteria, and uncover the following patterns. We observed that the maximal nutrient uptake rate and biomass yield were both decreasing functions of initial nutrient concentration. While a functional form for the relationship between biomass yield and initial nutrient concentration has been previously derived from first metabolic principles, here we also derive the form of the relationship between maximal nutrient uptake rate and initial nutrient concentration. Incorporating these two functions into a model of microbial growth allows for variable growth parameters and enables us to substantially improve predictions for microbial dynamics in a range of initial nutrient concentrations, compared to keeping growth parameters fixed.

## Author summary

Our ability to predict microbial population dynamics is of key importance for the fields of ecology, evolution, biotechnology, and public health. Yet, current mathematical models used to predict microbial growth have an inherent limitation. They are parameterised using empirical measurements of microbial growth performed at a single initial nutrient concentration. This overlooks the fact that in nature microbes face different levels of nutrient availability at all environmental scales: from glucose fluctuations in the blood of critically ill patients to dissolved organic carbon fluctuations in marine environments. Current literature overwhelmingly suggests that estimating growth parameters at a single initial nutrient concentration hampers the models from accurately capturing microbial dynamics when the environmental conditions change. Here we tackle this problem using an interplay between mathematical modelling and laboratory experiments spanning human fungal pathogens, common coliform bacteria, and baker’s yeast. We propose a modelling approach that incorporates growth parameters as a function of initial nutrient concentration. Importantly, we demonstrate that our approach performs significantly better at predicting microbial growth and the outcomes of between-species competition across different initial nutrient concentrations, compared to the classical models which assume fixed growth parameters.

## Introduction

Microbial communities shape the biogeochemistry of the planet [1, 2], the functioning of the ecosystem [3, 4], and the health of macro-organisms [5, 6]. Therefore, understanding and predicting microbial population dynamics is a key challenge for the fields of ecology, evolution, public health, and biotechnology. Mathematical models of microbial growth and metabolism form the foundation of many predictions regarding competition [7–9], metabolic interactions [10], cooperation [11–15], and diversification [16, 17] within microbial communities, as well as product formation and its optimisation for use in biotechnology [18, 19].

To grow and reproduce, microbes take up essential nutrients from the environment and this process can be represented mathematically in a number of ways involving different organisational scales. For example, genome-scale metabolic models use flux balance analysis [10, 12, 19–21] to provide testable predictions of metabolic activity at the whole genome scale. In contrast, ecological models aimed at predicting microbial population densities and frequencies within a community, often treat metabolism as a black box [8, 22, 23], while others incorporate an intermediate level of metabolic detail [13, 24, 25]. Regardless of their metabolic complexity, mathematical models generally assume that nutrient uptake is a saturating function of nutrient concentration. The parameters shaping such a function can subsequently be estimated by fitting a numerical solution of the mathematical model describing microbial population growth and/or nutrient uptake over time to empirically obtained data, typically for a selected initial nutrient concentration. However, this does not accurately reflect reality as in nature microbes regularly face changes in nutrient availability at all environmental scales [26]. For example, glucose concentrations in the blood of critically ill patients undergo substantial daily variations [27, 28], while rapid fluctuations in dissolved organic carbon are observed in marine environments [29]. Moreover, bacteria are known to experience changes in the ambient nutrient abundance, where periods of nutrient excess are followed by periods of its scarcity [30], or changes in the nutrient type [31].

Should we use fixed nutrient uptake parameters, estimated for a single specific initial nutrient concentration, to predict microbial dynamics in environments with different initial nutrient concentrations? Existing literature suggests not [32, 33]. In particular, a dynamic model whereby the maximal nutrient uptake rate increases monotonically as the external nutrient concentration decreases was successfully used to describe phytoplankton nutrient uptake [32]. In other studies, fitted values of the parameters associated with the nutrient uptake rate for both ecological [33] and genome-scale [12] models were found to be sensitive to the initial nutrient concentrations in the environment. Indeed, empirical studies conducted for a wide range of microbes growing on different nutrients demonstrate that nutrient uptake kinetics differ between the environments with high and low nutrient availability. Examples include nitrate, ammonia, and phosphorus uptake by various phytoplankton groups [34, 35], galactose accumulation by bacteria [36], and glucose uptake by yeast [37].

In general, changes in nutrient uptake kinetics can result from dynamic physiological responses to nutrient levels, detected by both extracellular membrane-localized receptors [38] and by intracellular sensing mechanisms [39]. Differing nutrient levels can trigger diverse physiological responses that alter uptake kinetics and growth. It can induce transcriptional responses that alter the types, quantities and activity of nutrient transporter proteins, such as the regulation of hexose transporters with different uptake kinetics in response to glucose concentration [40, 41]. It can also cause the removal and inactivation of transporters, modulate the affinity of specific transporters [42], or alter the expression of key metabolic genes, including those involved in glycolysis and gluconeogenesis [39]. Changes in nutrient uptake rates can even shift the metabolic pathways used by microbes, such as a shift from the more rapid and low ATP-yielding respiro-fermentation at high hexose uptake, to slower and higher ATP-yielding respiration at low hexose uptake [41, 43, 44]. Metabolic shifts, such as this, are one example of an underlying mechanism behind the widespread trade-off between growth rate (biomass per unit of time) and yield (biomass per unit of resource) that occurs throughout diverse microbial species [17, 22].

To examine the relationship between nutrient uptake parameters deployed in mathematical models and the initial nutrient concentration in the environment, we conduct comprehensive growth experiments for a range of micro-organisms including human fungal pathogens (*Candida albicans* and *Candida glabrata*), common coliform bacteria (*Escherichia coli*), and baker’s yeast (*Saccharomyces cerevisiae*) over a range of initial nutrient concentrations. Strikingly, for all organisms considered, we uncover a pattern whereby the value of the fitted parameter denoting the maximal nutrient uptake rate is a decreasing function of the initial nutrient concentration. Subsequently we derive the explicit form of this function from first metabolic principles. Although previous studies have suggested that the maximal nutrient uptake rate should not be considered constant under different environmental conditions [32, 35, 45, 46], to our knowledge an explicit mathematical form of such a function has not been previously derived.

Our study highlights the need for an alternative approach to modelling microbial growth. We propose that any new approach should take into account that the parameters describing nutrient uptake and growth kinetics vary along different environmental conditions. In particular, microbial biomass yield is a decreasing function of the initial nutrient concentrations according to the functional form derived previously [33], while the maximal nutrient uptake rate varies according to the functional form we propose here. Finally, we implement this proposal for a simple ecological model to show it can predict microbial growth and outcomes of competition between different species growing in a range of initial nutrient concentrations.

## Materials and methods

### A simple mathematical model of microbial growth

Motivated by well-established simple ecological models of microbial growth [8, 22, 23], we consider a microbe growing on a limiting nutrient N and assume that it takes up the nutrient and converts it into biomass B using a simple unbranched metabolic pathway [22, 23]. The rate of biomass production equals *Y* × *q*, where *q* denotes the rate of the pathway, while *Y* is the number of biomass units produced per unit of nutrient in the pathway. As in [22, 23], we make a simplifying assumption that the behaviour of the entire pathway can be modelled with Michaelis-Menten kinetics of a single reaction [47]. Therefore, *q* is the following function of the concentration of the limiting nutrient *N*:

$$\begin{array}{c} q\left(N\right)=\frac{V\times N}{K+N}\;, \end{array}$$ (1)

with *V* denoting the maximal rate of the pathway and *K* representing a half-saturation constant corresponding to the microbe. The pathway rate *q*(*N*) shows the rate at which product is formed which in this case is the same as the rate at which nutrient is consumed. Therefore, throughout this study we refer to *V* as the maximal rate of nutrient uptake and *K* as a half-saturation constant. The dynamics of growth begins with the introduction of the limiting nutrient (*N*) while observing the density of the microbe (*B*) in the environment. Subsequently, one season of growth of a length *T* is described by the following differential equation:

$$\begin{array}{c} \left\{ \begin{array}{c} \dot{N}\left(t\right)=-q\left(N\left(t\right)\right)\times B\left(t\right)\\ \dot{B}\left(t\right)=Y\times q\left(N\left(t\right)\right)\times B\left(t\right) \end{array}\;t\in \left[0,T\right]\;.\right. \end{array}$$ (2)

Note that the growth rate equals *Y* × *q*, where *q*(*N*(*t*)) is as in Eq (1), and thus the microbial growth depends not only on the parameters *Y*, *V*, and *K*, but also on the temporal dynamics of the nutrient concentration *N*(*t*).

The simple model (2) can easily be extended to incorporate a lag-phase growth term (see S1 Text, Appendix D for details).

### Growth experiments

Growth experiments of all species were performed in either duplicate (*Candida* species) or triplicate (*S. cerevisiae* and *E. coli* species) for each resource concentration, with each well in a microplate considered a replicate. Frozen stock cultures were revived on agar plates and starter cultures were grown overnight in appropriate liquid cultures. Growth measurements were conducted in liquid media in microplates that were sealed with a 50 *μ*m thick polyester film (VWR, UK) with holes pierced above each well with a sterile needle for gas exchange. All microplates were shaken during the growth period to enhance diffusive gas exchange and ensure even distribution of well contents. For the yeast species, 48-well plates were used to enhance the mixing, since 96-well plates can be problematic for achieving sufficient oxygen supply [48] and sustained exponential growth of microbes [49]. Wells with uninoculated media were included as blank measurements and contamination checks. For all species, growth was measured as optical density in a microplate reader, which was converted to cell density using calibrations of known densities from plating. Species-specific protocols are detailed in the following sections. The raw data for all experiments from this study is available at DOI: https://doi.org/.6084/9.figshare.13193600.v3.

#### Growth experiments with *Candida* species

The strain of *C. albicans* ACT1-GFP was used, which is the SBC153 strain tagged with GFP at the ACT1 locus using a nourseothricin resistance cassette. The wild-type reference strain of *C. glabrata* ATCC2001 was used. Overnight cultures of either *C. albicans* or *C. glabrata* were prepared by inoculating a single colony into 5 ml of YPD medium (except for one *C. glabrata* experiment where the overnight was performed in SC 2%) and incubating at 30°C and 350 rpm for 16–18 hours. The cells were washed in water, counted in a haemocytometer and resuspended in SC (6.9 g/l Yeast Nitrogen Base w/o amino acids, 790 mg/l complete supplement mixture (Formedium, UK)) containing glucose to the desired concentration. For the growth experiments in S1 Text, Appendix C, C1 Fig, nutrient enriched media at 1.8 × the concentration of the control medium was prepared with a final concentration of 12.4 g/l Yeast Nitrogen Base and 1422 mg/l complete supplement mixture and glucose to the desired concentration. pH buffered medium contained the addition of 0.1M potassium phosphate (pH 6). The suspension of cells was added to media in the wells of a 48-well suspension culture plates (640 *μ*l per well (Greiner Bio-One)) in order to achieve 640 *μ*l of 1 × 10^7^ cell/ml in glucose concentrations in the range (0.025–4% (w/v)). Plates were incubated at 30°C with 510 rpm orbital shaking and growth monitored by measuring optical density (OD) at 620 nm in a Spark 10 M (Tecan) microplate reader. OD was converted into cell density (CFU) based on the calibrations in S1 Text, Appendix B, B8 Fig (a) and (b).

#### Growth experiments with *S. cerevisiae*

Growth assays of *S. cerevisiae* were conducted with strain CEN.PK2-1C grown on SC media with sucrose at different concentrations (4%, 1%, 0.25%, 0.0625% (w/v)). The verifications of metabolic inefficiencies in S1 Text, Appendix C, C1 Fig were conducted with an engineered strain of *S. cerevisiae* (TM6*). This strain has reduced hexose transport capabilities that causes it to have fully respiratory metabolism, which has higher efficiency in terms of ATP yield than the wild-type that has respiro-fermentative metabolism [43]. Overnight cultures were established in 5 ml SC with 1% (w/v) glucose and grown overnight at 30°C with 180 rpm shaking. Cells were washed and inoculated into fresh SC media containing sucrose at the specified concentrations at an initial density of 500 CFU/*μ*l which was determined based on spectrophotometer measurements calibrated to known densities. Cultures were inoculated into 48-well suspension culture plates (640 *μ*l per well (Greiner Bio-One)) and incubated in a FLUOstar Omega microplate reader (BMG) at 30°C with 700 rpm orbital shaking. Population density was measured by OD 620 nm approximately every 15 minutes and converted to cell density (CFU) based on previously used calibrations [14].

#### Growth experiments with *E. coli*

*E. coli* MG1655 was grown in DM media (potassium phosphate dibasic 7 g/l, potassium phosphate monobasic 2 g/l, ammonium sulfate 1 g/l, sodium citrate 0.5 g/l, 1M magnesium sulfate 1 ml/l, 0.02% thiamine 1 ml/l, 1.5 mM calcium chloride (Sigma-Aldrich UK)) supplemented with 0.1% casamino acids and a range of glucose concentrations (0.05%, 0.1%, 0.2%, 0.4% (w/v)). Overnight cultures were established in 10 ml 0.1% glucose DM media and incubated for 20–24 hours at 30°C with 160 rpm shaking. pH was recorded at the start of the overnight culture (0 hours) and at the end (24 hours) to observe any changes due to metabolic products of *E. coli* growth. pH of growth culture was observed as 7.0 at 0 hours and 6.9 at 24 hours, indicating no substantial acidification of growth media. The concentrations of phosphorus and nitrogen used in the experiments are similar to those previously described as abundant [50], indicating that they are not limiting substrates in our experiment. Cells were inoculated into fresh media supplemented with the specified concentration of glucose at 10^6^ CFU/ml, determined by spectrophotometer measurements calibrated to known densities. Cultures were transferred into clear, flat bottomed 96-well plates (Griener Bio-One, UK) and incubated in a Biotek ELx808 microplate reader (Agilent, USA) at 30°C for 24 hours with 5 minutes of linear shaking at medium intensity prior to each read. Density was measured by 600 nm every 20 minutes and converted into cell density (CFU) based on the calibrations in S1 Text, Appendix B, B8 Fig (c).

## Results

### Dependence of growth model parameters on the initial nutrient concentration

Our simple ecological model of microbial growth (2) contains three free parameters (*V*, *K*, *Y*) that we estimate for each microbial species by fitting numerical solutions of the model (2) to the experimentally obtained data using four microbial species and a range of initial nutrient concentrations. In particular, we consider: *C. albicans* growing on glucose (see S1 Text, Appendix B, B1 Fig and S1 Text, Appendix A, A1 Table), *C. glabrata* growing on glucose (see S1 Text, Appendix B, B2 Fig and S1 Text, Appendix A, A2 Table), *S. cerevisiae* growing on sucrose (see S1 Text, Appendix B, B3 Fig and S1 Text, Appendix A, A3 Table), and *E. coli* growing on glucose (see S1 Text, Appendix B, B4 Fig and S1 Text, Appendix A, A4 Table).

**Fig 1:**
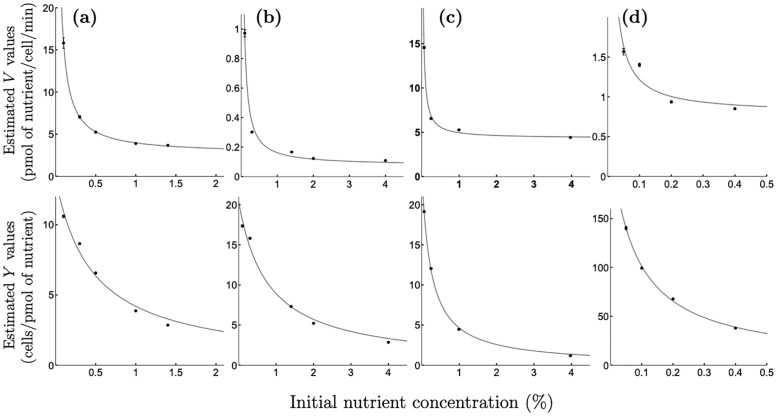
Relationships ‘maximal uptake rate vs initial nutrient concentration’ and ‘biomass yield vs initial nutrient concentration’. Relationships between the maximal uptake rate *V* and the initial nutrient concentration *N*_0_ (top panels) and between the yield *Y* and the initial nutrient concentration *N*_0_ (bottom panels) observed for (a) *C. albicans* growing on glucose; (b) *C. glabrata* growing on glucose; (c) *S. cerevisiae* growing on sucrose; (d) *E. coli* growing on glucose. Dots with error bars (estimated values ± SE, where SE might be obscured by values) represent optimal estimates for parameters *V* (top panels) and *Y* (bottom panels) obtained by fitting the model (2) to the experimental data on growth (see S1 Text, Appendix B, B1 Fig—B4 Fig and S1 Text, Appendix A, A1 Table—A4 Table for growth data corresponding to (a)—(d), respectively). Solid lines are the optimal non-linear least-squares fits of the function (8) (top panels) and of the function (3) (bottom panels) to the plotted data. The functional forms of *Y*(*N*_0_) and *V*(*N*_0_) are not sensitive to media enrichment or acidification (see S1 Text, Appendix C for details) nor to the addition of the lag-phase growth term into the model (2) (as described in S1 Text, Appendix D).

While for all species tested, the estimated value of *K* did not depend on the initial nutrient concentration in the environment (see S1 Text, Appendix A, A1 Table—A4 Table), we observe that the estimated values of *V* and *Y* decrease as the initial nutrient concentration increases (see Fig 1).

It has been shown in [33] that the relationship between the biomass yield and the extracellular nutrient concentration takes on the following mathematical form:

$$\begin{array}{c} Y\left(N_{0}\right)=Y_{\text{hi}}\frac{1}{1+pN_{0}}+Y_{\text{lo}}\frac{pN_{0}}{1+pN_{0}}\;, \end{array}$$ (3)

where *Y*_hi_ is the highest possible yield that can be achieved at low initial nutrient concentrations, while *Y*_lo_ represents the biomass yield when there is an extracellular nutrient excess, and *p* indicates a phenotype that controls the rate of change in the biomass yield due to the changes in ambient conditions. We observe that the functional form (3) fits the data for all four microbial species we consider, shown in Fig 1 (bottom row).

#### Functional forms for *V* and *K*

In order to derive functional forms for *V* and *K* to be used in our ecological model Eq (2), we develop an enzyme-kinetic model based on the following assumptions. Nutrient uptake and its conversion into biomass is modelled as a simple unbranched metabolic pathway represented by a single reaction. Moreover, we consider a medium with a bulk nutrient N_0_, and a cell in this medium which can capture the nutrient from the environment and transfer it further into the cytoplasm by means of its uptake sites, or transporters, E. We also assume that the uptake sites are immobilized (attached to the cell’s membrane). It is possible, therefore, that a boundary layer surrounds the cell with a local nutrient concentration [N_loc_] that is smaller than [N_0_] due to the differences between the diffusive flux of nutrient from the bulk to the cell and the cell nutrient consumption flux. For the sake of simplicity and to align with the experimental conditions used, we hereafter assume the case of fast diffusion, which means that [N_loc_] ≈ [N_0_].

The nutrient uptake process in such a system can then be described as the following open enzymatic reaction scheme, which is motivated by the one proposed by Bonachela et al. (Fig 1 in the main text in [32]):

$$\begin{array}{cc} \;\;\;\; & {\text{E}}_{\text{f}}+{\text{N}}_{\text{loc}}\overset{k_{1}}{⟶}{\text{EN}}_{\text{loc}}\overset{k_{2}}{⟶}{\text{N}}_{\text{i}}+{\text{E}}_{\text{f}}\;,\\ & ↑\\ & \Phi_{\text{E}} \end{array}$$

where a local nutrient molecule N_loc_ encounters and binds an unoccupied enzyme E_f_ to form an enzyme-nutrient complex EN_loc_, and is subsequently incorporated as an internal nutrient N_i_ into the cell’s cytoplasm. Here, Φ_E_ denotes the flow of new uptake sites synthesized by the cell, which essentially represents the ability of the cell to regulate the number of uptake sites depending on the external conditions.

The following mathematical model describes the reactions in this pathway:

$$\left\{ \begin{array}{ll} d[{\text{E}}_{\text{f}}]/dt=-k_{1}[{\text{E}}_{\text{f}}][{\text{N}}_{\text{loc}}]+k_{2}[{\text{EN}}_{\text{loc}}]+\Phi_{\text{E}} & \left(4\text{a}\right)\\ d[{\text{N}}_{\text{loc}}]/dt=-k_{1}[{\text{E}}_{\text{f}}][{\text{N}}_{\text{loc}}] & \left(4\text{b}\right)\\ d[{\text{EN}}_{\text{loc}}]/dt=k_{1}[{\text{E}}_{\text{f}}][{\text{N}}_{\text{loc}}]-k_{2}[{\text{EN}}_{\text{loc}}] & \left(4\text{c}\right)\\ d[{\text{N}}_{\text{i}}]/dt=k_{2}[{\text{EN}}_{\text{loc}}]. & \left(4\text{d}\right) \end{array}\right.$$

If we assume that the time the cell needs to capture the first nutrient molecule at the beginning of the uptake process is small, then the number of occupied uptake sites [EN_loc_] can be considered approximately constant [32, 51], so that *d*[EN_loc_]/*dt* ≈ 0, whence from Eq (4c) we have:

$$\begin{array}{c} \left[{\text{EN}}_{\text{loc}}\right]=\frac{k_{1}\left[\text{E}\right]\left[{\text{N}}_{\text{loc}}\right]}{k_{2}+k_{1}\left[{\text{N}}_{\text{loc}}\right]}\;, \end{array}$$ (5)

where [E] = [E_f_] + [EN_loc_] is the total number of uptake sites. Combining this equation with the definitions *U* = *k*_2_[EN_loc_], *V*_max_ = *k*_2_[E], *K*_m_ = *k*_2_/*k*_1_, we derive the Michaelis-Menten equation as follows:

$$\begin{array}{c} U=\frac{V_{\text{max}}\left[{\text{N}}_{\text{loc}}\right]}{K_{\text{m}}+\left[{\text{N}}_{\text{loc}}\right]}\;. \end{array}$$ (6)

The total number of uptake sites [E] is not constant as it was in a classical form of the Michaelis-Menten equation [47], since from Eq (4c) and (4a) we can see that *d*[E]/*dt* = *d*[E_f_]/*dt* + *d*[EN_loc_]/*dt* = Φ_E_, which means that the cell can regulate the number of its uptake sites depending on the external (and/or internal) conditions. This transforms the static parameter *V*_max_ from the Michaelis-Menten equation into the dynamic kinetic parameter *V*_max_ from Eq (6), and here we aim to derive its dependence on the bulk nutrient concentration [N_0_] explicitly.

From Eq (5) we have:

$$\begin{array}{c} \left[\text{E}\right]=\left[{\text{EN}}_{\text{loc}}\right](\frac{k_{2}/k_{1}}{\left[{\text{N}}_{\text{loc}}\right]}+1)\;. \end{array}$$ (7)

By combining this with the definition *V*_max_ = *k*_2_[E], we arrive at the following relationships between the maximal uptake rate *V*_max_ and the local nutrient concentration [N_loc_]:

$$\begin{array}{c} V_{\text{max}}=p_{v}+\frac{q_{v}}{\left[{\text{N}}_{\text{loc}}\right]}\;, \end{array}$$

with parameters *p*_*v*_ = *k*_2_[EN_loc_] and $q_{v}=\frac{{\left(k_{2}\right)}^{2}}{k_{1}}\left[{\text{EN}}_{\text{loc}}\right]$.

By assuming the case of fast diffusion, so that [N_loc_] ≈ [N_0_], and using our previous notation *N*_0_ instead of [N_0_] for the bulk nutrient concentration, we can rewrite the obtained equation as follows:

$$\begin{array}{c} V_{\text{max}}\left(N_{0}\right)=p_{v}+\frac{q_{v}}{N_{0}}\;. \end{array}$$ (8)

Thus we can conclude that Eq (6) takes on a classical form of the Michaelis-Menten equation with the dynamic parameter *V*_max_ defined by Eq (8) and the constant parameter

$$\begin{array}{c} K_{\text{m}}\left(N_{0}\right)=c\;, \end{array}$$ (9)

where *c* denotes a constant.

Motivated by the above enzyme-kinetic derivations, we will hereafter use the functions (8) and (3) in our model (2) as analytical representations of the relationship ‘maximal nutrient uptake rate (*V*) vs initial nutrient concentration (*N*_0_)’ and ‘biomass yield (*Y*) vs initial nutrient concentration (*N*_0_)’, respectively, and assume that the half-saturation constant (*K*) does not depend on the initial nutrient concentration.

### Predicting microbial growth and competition outcomes across a range of initial nutrient concentrations

We propose the following approach to predict microbial growth across a specific range of initial nutrient concentrations [*a*, *b*].

Step 1: Collect experimental growth data for at least three initial nutrient concentrations *N*_0,*i*_ ∈ [*a*, *b*](*i* = 1..3). Note, three observations {*N*_0,*i*_, *Y*_*i*_}(*i* = 1..3) are the minimum required to fit Eq (3) which contains three free parameters.
Step 2: For each of these initial nutrient concentrations *N*_0,*i*_, estimate *V*_*i*_, *K*_*i*_, and *Y*_*i*_ by fitting numerical solutions of the model (2) to the experimentally obtained data in Step 1.
Step 3: Derive the forms for *Y*(*N*_0_), *V*(*N*_0_), and *K*(*N*_0_) by fitting Eqs (3), (8), and (9) to the data {*N*_0,*i*_, *Y*_*i*_}, {*N*_0,*i*_, *V*_*i*_}, and {*N*_0,*i*_, *K*_*i*_}, respectively, obtained in Step 2.
Step 4: Predict microbial growth at a given initial nutrient concentration $N_{0}^{*}\in [a,b]$ by carrying out a numerical simulation of the model (2) with the parameters $Y(N_{0}^{*})$, $V(N_{0}^{*})$, and $K(N_{0}^{*})$ derived in Step 3.

We illustrate the proposed approach with the two following examples. First, we predict growth dynamics of a microbe (i.e. *C. glabrata* species growing on glucose), and second, outcomes of competition between two micro-organisms (i.e. *C. albicans* and *C. glabrata* species competing for glucose).

#### Example 1: Predicting microbial growth

We seek to predict the growth of a human fungal pathogen *C. glabrata* across the following range of glucose concentration [*a*, *b*] = [0.025%, 2%].

**Fig 2:**
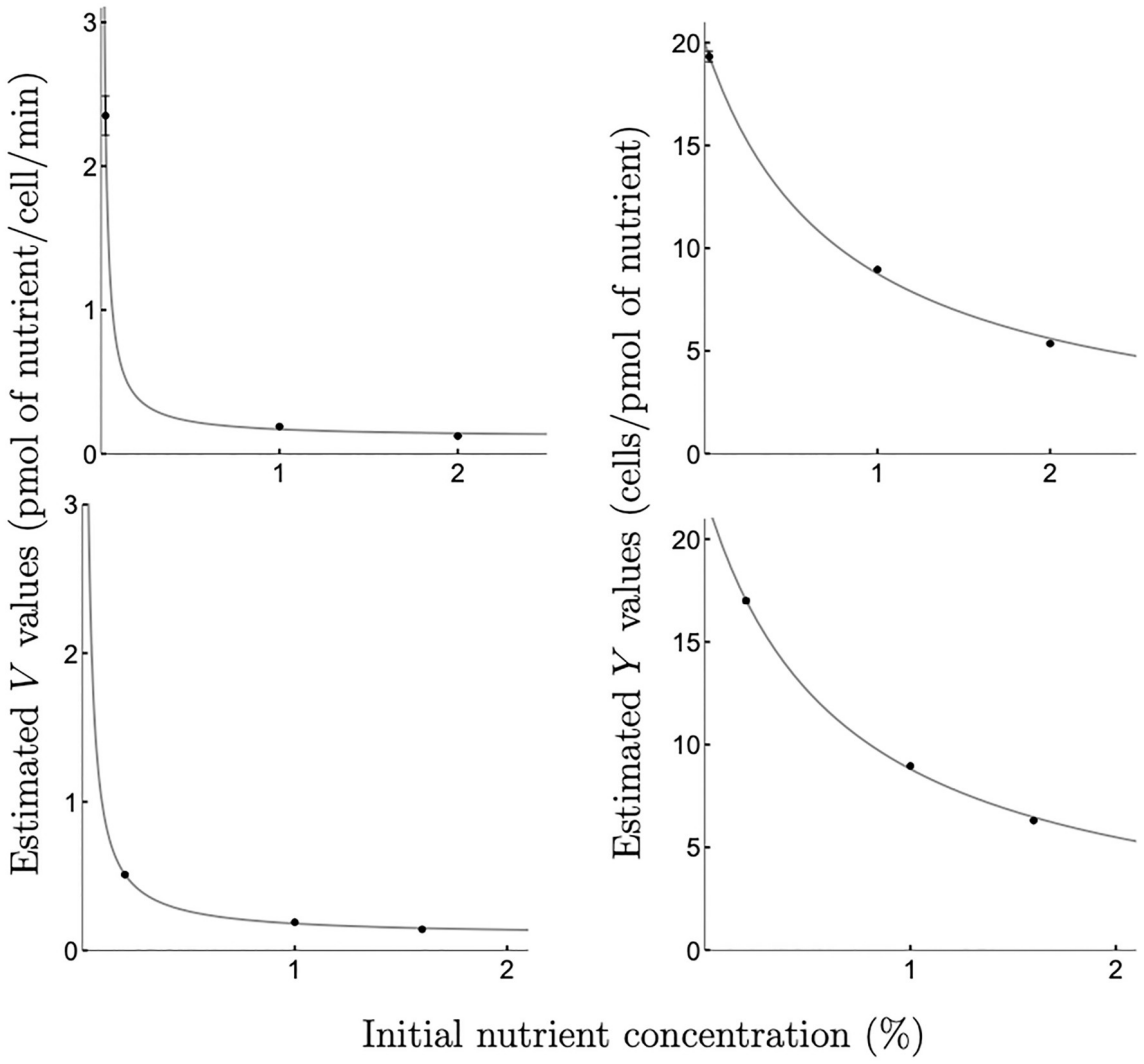
Relationships ‘maximal uptake rate vs initial nutrient concentration’ and ‘biomass yield vs initial nutrient concentration’ observed for *C. glabrata* growing on glucose. Relationships between the maximal uptake rate *V* and the initial nutrient concentration *N*_0_ (left panels) and between the yield *Y* and the initial nutrient concentration *N*_0_ (right panels) observed for *C. glabrata* growing on glucose. Dots with error bars (estimated values ± SE, where SE might be obscured by values) represent optimal estimates for parameters *V*_*i*_ with *i* = 1..3 (left panels) and *Y*_*i*_ with *i* = 1..3 (right panels). These were obtained by fitting the model (2) to the experimental data on growth at the following initial glucose concentrations *N*_0,*i*_ (*i* = 1..3): 0.025%, 1%, 2% (top panels, correspond to growth data shown in S1 Text, Appendix B, B5 Fig and S1 Text, Appendix A, A5 Table) and at the following initial glucose concentrations *N*_0,*i*_ (*i* = 1..3): 0.2%, 1%, 1.6% (bottom panels, correspond to growth data shown in S1 Text, Appendix B, B6 Fig and S1 Text, Appendix A, A6 Table). Solid lines are the optimal non-linear least-squares fit of the function (8) (left panels) and of the function (3) (right panels) to the plotted data. Note that we refer to *V*_*i*_ and *Y*_*i*_ in the context of Example 1, while the same parameters were labelled $V_{G}^{i}$ and $Y_{\text{G}}^{i}$ in Example 2, with the subscript G distinguishing *C. glabrata* data used to parameterise the model of competition with *C. albicans*.

**Fig 3:**
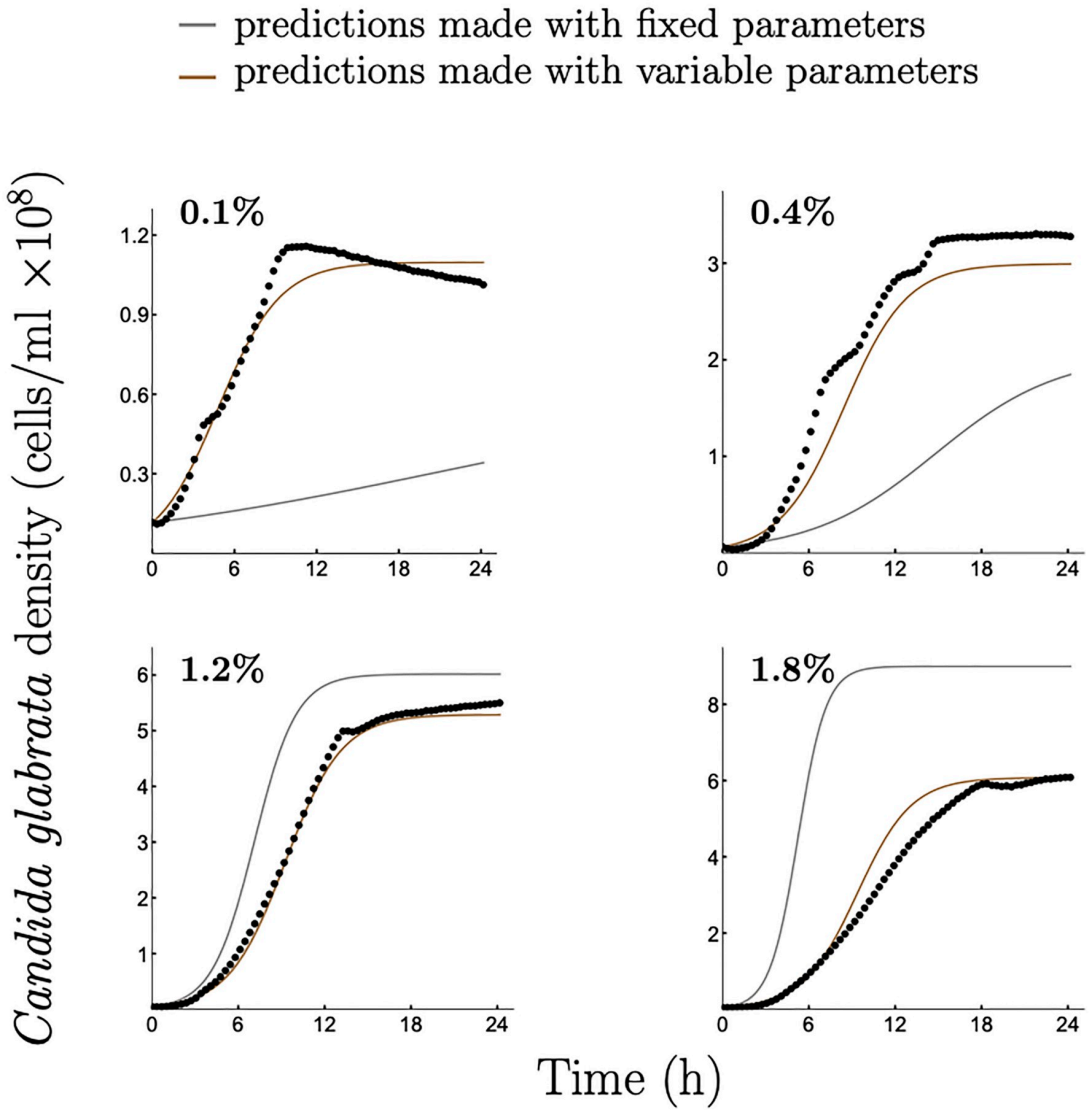
Comparison of growth predictions made by two modelling approaches, one using fixed and the other variable growth kinetic parameters. Experimentally obtained growth data for *C. glabrata* at different glucose concentrations (0.1%; 0.4%; 1.2%; 1.8%) are denoted by dots. Gray curves represent predictions of a model with fixed growth parameters estimated at the 1% initial glucose concentration (see S1 Text, Appendix B, B5 Fig (b) for growth data at 1% glucose). Brown curves represent predictions of a model with variable growth parameters as proposed in this study with [*a*, *b*] = [0.025%, 2%] in Step 1. To quantify the accuracy of the predictions made by the two approaches the Root Mean Square Error (RMSE) was calculated (see S1 Text, Appendix A, A8 Table for more details). Note, y-axis scale changes between panels.

Step 1: Obtain experimental growth data for the following chosen initial glucose concentrations {*N*_0,*i*_ |_*i*=1..3_} = {0.025%, 1%, 2%} (shown in S1 Text, Appendix B, B5 Fig).
Step 2: Estimate *V*_*i*_, *K*_*i*_, and *Y*_*i*_ for each initial glucose concentration *N*_0,*i*_ (*i* = 1..3) by fitting numerical solutions of the model (2) to the experimental growth data. The parameter estimates are shown in S1 Text, Appendix A, A5 Table.
Step 3: Derive the forms for *Y*(*N*_0_), *V*(*N*_0_), and *K*(*N*_0_) by fitting Eqs (3), (8) and (9) to the data {*Y*_*i*_, *i* = 1..3}, {*V*_*i*_, *i* = 1..3}, and {*K*_*i*_, *i* = 1..3}, respectively, obtained in Step 2 (see Fig 2, top panels).
Step 4: To predict microbial growth at an initial glucose concentration $N_{0}^{*}\in \left[0.025\%,2\%\right]$ different from the initial concentrations chosen in Step 1, say $N_{0}^{*}=0.1\%$, carry out a numerical simulation of the model (2) for parameters $Y\left(N_{0}^{*}\right),\;V\left(N_{0}^{*}\right)$, and $K(N_{0}^{*})$ derived in Step 3 (Fig 3, top left panel). Repeat the same procedure for a variety of different initial nutrient concentrations $\left\{N_{0,i}^{*}{\;|}_{i=1..3}\}=\{0.4\%,1.2\%,1.8\%\right\}$. Results are shown in Fig 3.

The resulting growth predictions perform better for a range of initial glucose concentrations than the predictions generated by a commonly used modelling approach where fixed growth kinetics parameters are estimated from a single resource concentration (Fig 3). The latter fits growth parameters to an arbitrarily chosen initial glucose concentration and uses those parameters to numerically simulate microbial growth at different glucose concentrations [19]. The statistical comparisons of the two approaches are summarised in S1 Text, Appendix A, A8 Table.

#### The choice of initial nutrient concentrations

**Fig 4:**
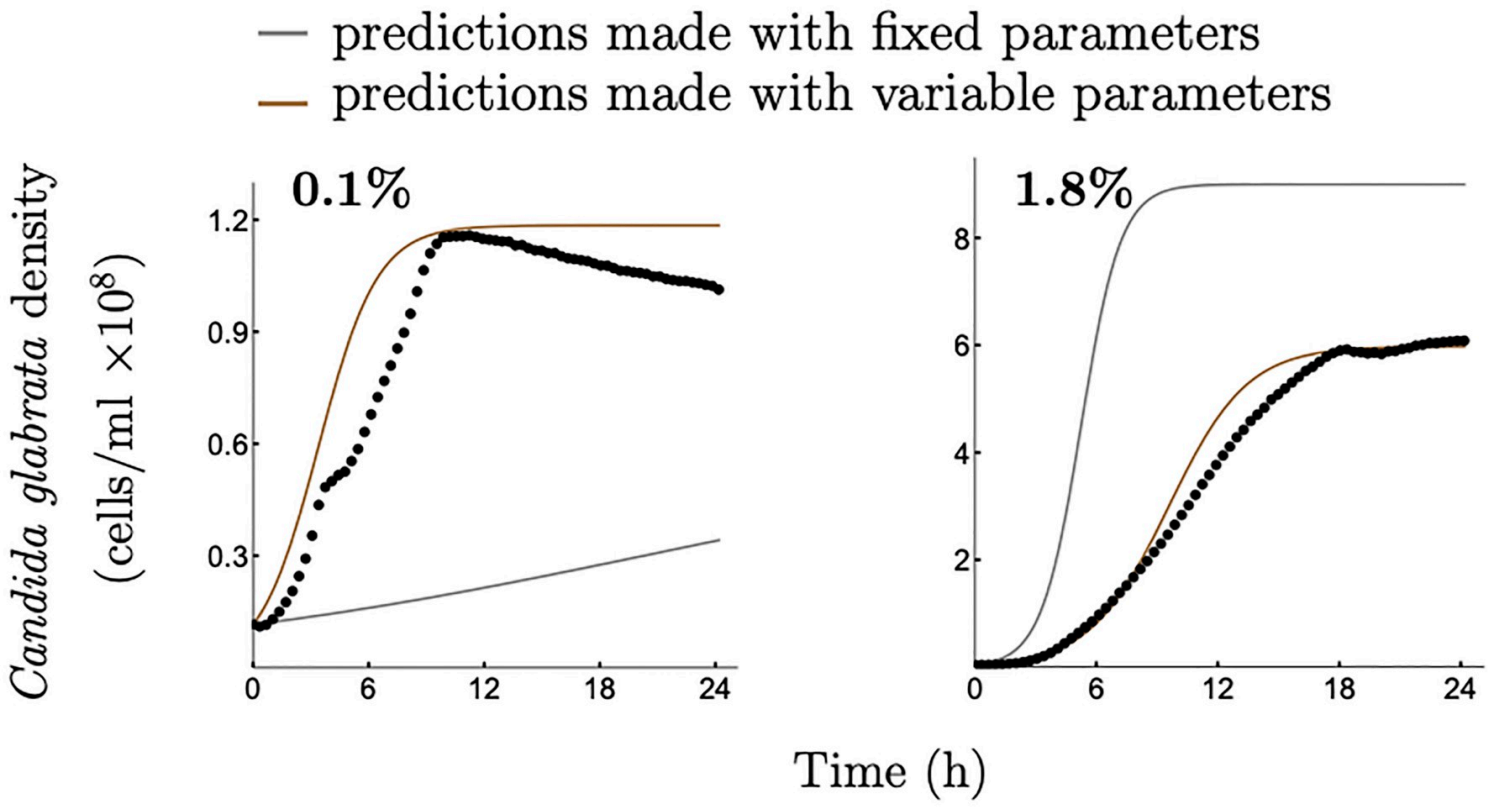
Comparison of growth predictions made by two modelling approaches, one using fixed and the other variable growth kinetic parameters. Experimentally obtained growth data for *C. glabrata* at different glucose concentrations (0.1%; 1.8%) are denoted by dots. Gray curves represent predictions of a model with fixed growth parameters estimated at the 1% initial glucose concentration (see S1 Text, Appendix B, B5 Fig (b) for growth data at 1% glucose). Brown curves represent predictions of a model with variable growth parameters as proposed in this study with [*a*, *b*] = [0.2%, 1.6%] in Step 1. To quantify the accuracy of the predictions made by the two approaches the Root Mean Square Error (RMSE) was calculated (see S1 Text, Appendix A, A9 Table for more details). Note, y-axis scale changes between panels.

Next we ask, how well does our approach predict microbial growth at concentrations outside the [*a*, *b*] interval? To this end, we choose [*a*, *b*] = [0.2%, 1.6%] (see Fig 2, bottom panels) and seek to predict microbial growth at 0.1% and 1.8% initial glucose concentrations—the values lying outside of the chosen interval. Following the Steps 1–4, as above, we show that our method can again outperform the fixed growth parameters approach (see Fig 4 together with S1 Text, Appendix A, A9 Table).

#### Example 2: Predicting the outcomes of microbial competition

Next, we seek to predict the outcome of competition between two microbial species using the new approach proposed here and compare it to the predictions generated by a common approach that uses growth parameters estimated from a single initial nutrient concentration [7, 9]. To this end, we consider a microbial community consisting of *C. albicans* and *C. glabrata* competing for limiting glucose in the environment. The competition dynamics during a single growing season of length *T* can be described by extending the model (2) as follows:

$$\begin{array}{c} \left\{ \begin{array}{c} \dot{N}\left(t\right)=-\sum_{k\in \{\text{A,G}\}}q_{k}\left(N\left(t\right)\right)\times B_{k}\left(t\right)\\ {\dot{B}}_{k}\left(t\right)=Y_{k}\times q_{k}\left(N\left(t\right)\right)\times B_{k}\left(t\right) \end{array}\;t\in \left[0,T\right]\;,\right. \end{array}$$ (10)

with *q*_*k*_(*N*) = *V*_*k*_ × *N*/(*K*_*k*_ + *N*), *k* ∈ {A, G}, and indices A and G corresponding to the species *C. albicans* and *C. glabrata*, respectively. To explore competition dynamics over multiple seasons, at the end of each season, a fixed number of cells is transferred to a new environment containing replenished growth medium with the limiting glucose at the same concentration at the start of each season. This setup mimics the experimental batch culture conditions where there is no constant inflow of nutrients.

**Fig 5:**
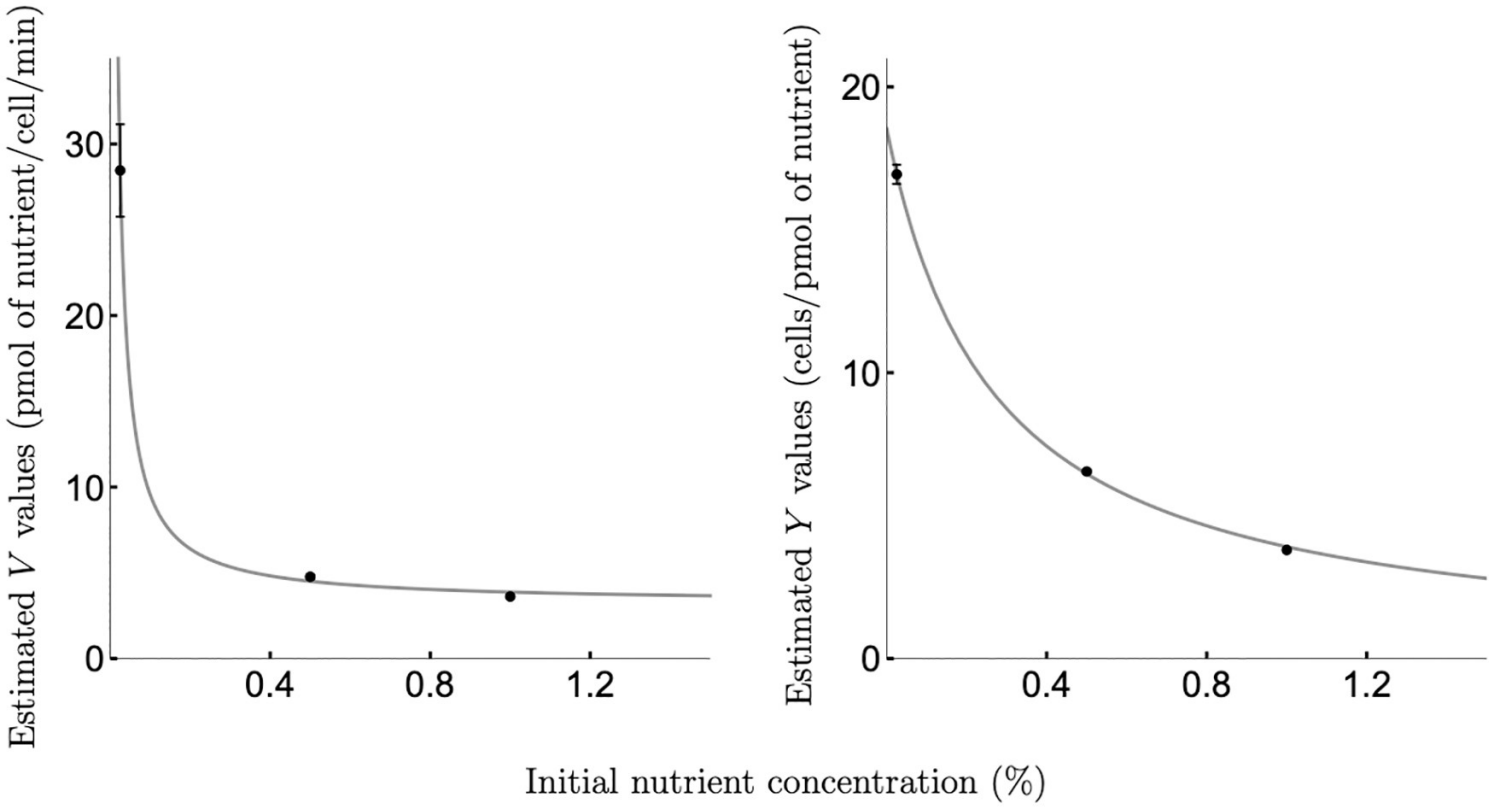
Relationships ‘maximal uptake rate vs initial nutrient concentration’ and ‘biomass yield vs initial nutrient concentration’ observed for *C. albicans* growing on glucose. *Left*: relationships between the maximal uptake rate parameter *V* and the initial nutrient concentration *N*_0_ observed for *C. albicans* growing on glucose. Dots with error bars (estimated values ± SE, where SE might be obscured by values) represent optimal estimates for maximal uptake rate parameters $V_{\text{A}}^{i}\;\left(i=1..3\right)$ obtained by fitting the model (2) to the experimental data on growth at the following initial glucose concentrations *N*_0,*i*_ (*i* = 1..3): 0.025%, 0.5%, 1% (see S1 Text, Appendix B, B7 Fig and S1 Text, Appendix A, A7 Table), and a solid line is the optimal non-linear least-squares fit of the function (8) to the plotted data. *Right*: relationships between the yield parameter *Y* and the initial nutrient concentration *N*_0_ observed for *C. albicans* growing on glucose. Dots with error bars (estimated values ± SE, where SE might be obscured by values) represent optimal estimates for yield parameters $Y_{\text{A}}^{i}\;\left(i=1..3\right)$ obtained by fitting the model (2) to the experimental data on growth at the following initial glucose concentrations *N*_0,*i*_ (*i* = 1..3): 0.025%, 0.5%, 1% (see S1 Text, Appendix B, B7 Fig and S1 Text, Appendix A, A7 Table), and a solid line is the optimal non-linear least-squares fit of the function (3) to the plotted data.

**Fig 6:**
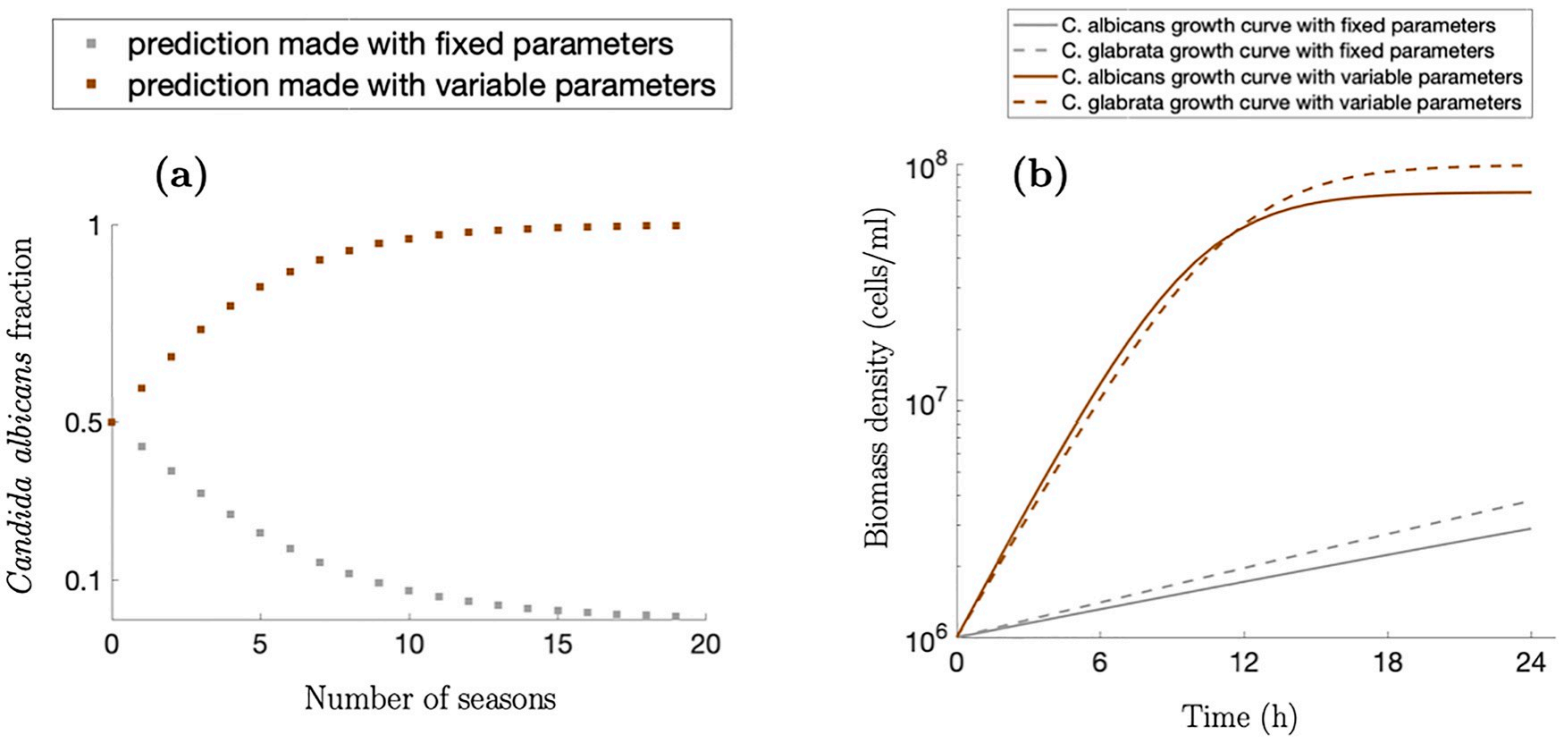
**(a) Predicted multi-season competition outcomes**. Modelled dynamics of the final *C. albicans* fraction in the microbial community consisting of *C. albicans* and *C. glabrata* in competition for a glucose at 0.1% glucose over 20 seasons of competition, as predicted by model (10). Each season’s length is 24 hours, the initial population density is 10^6^ cells/ml, and the initial fraction of *C. albicans* in the community is 0.5. Brown markers show predictions based on the approach proposed in this study. Gray markers are predictions obtained by using growth parameters estimated at 1% of glucose for each species (see S1 Text, Appendix B, B7 Fig (c) for *C. albicans* and S1 Text, Appendix B, B5 Fig (b) for *C. glabrata*). **(b) Theoretical growth of *C. albicans* and *C. glabrata* on glucose**. Theoretical growth dynamics for *C. albicans* (solid curves) and *C. glabrata* (dashed curves) in isolation, in 0.1% glucose over 24 hours. The simulation of the model (2) from the main text was carried out with variable growth parameters (brown curves) and growth parameters fixed at 1% glucose (gray curves).

To predict the competition outcomes over multiple seasons, we carry out Steps 1–4 as follows:

Step 1: We choose the following initial glucose concentrations $\left\{N_{0,i}^{\text{A}}{\;|}_{i=1..3}\}=\{0.025\%,0.5\%,1\%\right\}$ for *C. albicans*, with experimental growth data shown in S1 Text, Appendix B, B7 Fig; and $\left\{N_{0,i}^{\text{G}}{\;|}_{i=1..3}\}=\{0.025\%,1\%,2\%\right\}$ for *C. glabrata*, with experimental growth data shown in S1 Text, Appendix B, B5 Fig.
Step 2: For each species *k* ∈ {A, G}, at each of the initial glucose concentrations $N_{0,i}^{k}\;\left(i=1..3\right)$ we estimate $V_{k}^{i},\;K_{k}^{i}$, and $Y_{k}^{i}$ by fitting numerical solutions of the model (2) to the experimentally obtained data shown in S1 Text, Appendix B, B7 Fig and B5 Fig for *C. albicans* and *C. glabrata*, respectively. The parameter estimates are shown in S1 Text, Appendix A, A7 Table for *C. albicans* and in S1 Text, Appendix A, A5 Table for *C. glabrata*.
Step 3: For each species *k* ∈ {A, G}, we derive the forms for *Y*_*k*_(*N*_0_), *V*_*k*_(*N*_0_), and *K*_*k*_(*N*_0_) by fitting Eqs (3), (8) and (9) to the data $\left\{Y_{\text{k}}^{i}\;,\;i=1..3\right\}$, $\left\{V_{\text{k}}^{i}\;,\;i=1..3\right\}$ and $\left\{K_{\text{k}}^{i}\;,\;i=1..3\right\}$, respectively, obtained in Step 2 (see Fig 2, top panels, for *C. glabrata* and Fig 5 for *C. albicans*).
Step 4: To predict multi-season competition outcomes where each season begins with glucose concentration $N_{0}^{*}=0.1\%$, we carry out multi-season numerical simulation of the competition model (10) for parameters $Y_{k}\left(N_{0}^{*}\right),\;V_{k}\left(N_{0}^{*}\right)$, and $K_{k}\left(N_{0}^{*}\right)$ derived in Step 3 (Fig 6(a), brown markers).

The competition outcome between two species predicted by our approach (Fig 6(a), brown markers) differs critically from the outcome predicted by a competition model which uses fixed kinetic parameters (Fig 6(a), gray markers). The latter fits growth parameters to an arbitrarily chosen initial nutrient concentration (here taken as 1%). Subsequently, those parameters are used to numerically simulate the competition model (10) over multiple seasons, where each season is initiated at 0.1% glucose, predicting that *C. albicans* loses the competition (Fig 6(a), gray markers). In contrast, our approach is consistent with previous empirical studies (see Fig 1 (b) in [7]) predicting that *C. albicans* wins at 0.1% glucose concentration (Fig 6(a), brown markers).

Previous empirical studies observed that *C. glabrata* outcompetes *C. albicans* at high glucose concentrations [7]. Here we illustrate that estimating kinetic parameters at a high glucose concentration cannot accurately predict competition outcomes at low glucose concentrations as it gives an unfair growth advantage to *C. glabrata* over *C. albicans* (Fig 6(b)). In contrast, our approach incorporates the empirically observed variability in growth kinetics parameters, which gives *C. albicans* an initial growth advantage over *C. glabrata* (see Fig 6(b) for an illustration) enabling its competitive dominance in low glucose environments. The difference in competition outcomes between environments with different initial nutrient conditions is indicative of the metabolic differences between *Candida* species, which are thought to have diverged about 300 million years ago [52]. For instance, *C. albicans* and *C. glabrata* possess different repertoires of identified hexose transporters and have different glucose sensors that respond differently to low glucose environments. In particular, *C. albicans* lacks a low-affinity glucose sensor that *C. glabrata* possesses. This adaptation is thought to be the result of their different lifestyles because *C. albicans* has predominantly co-evolved with a human host, where high levels of glucose are generally rarer, whereas *C. glabrata* is more closely related to *S. cerevisiae* and so is thought to have experienced high and low glucose levels in nature [53, 54]. Moreover, Crabtree-positive yeasts, such as *C. glabrata* and *S. cerevisiae*, use fermentation even in the presence of oxygen, a strategy thought to have evolved in high sugar environments around the time that fruiting plants emerged [55, 56]. This is also consistent with recent observations that *C. glabrata* is more frequently isolated from infections of diabetic patients with high blood sugar levels [57, 58]. The above arguments could explain why *C. albicans* might be better adapted to low glucose environments and why *C. glabrata* has elevated growth rates in high glucose environments, as we observed for our predicted competition outcomes (Fig 6).

## Discussion

Here we provide a simple approach for predicting microbial growth dynamics in environments with different nutrient concentrations. This is important as current mathematical models estimate key microbial growth parameters in a single fixed initial nutrient condition [7, 8, 10, 12, 19] which can prevent them from accurately capturing growth dynamics when the initial nutrient conditions change.

In particular, the process of nutrient uptake required for microbial growth is classically represented in mathematical models as a saturating function of the nutrient concentration [22, 32]. The parameters shaping such a saturating function are fitted to empirically obtained data, typically for a single initial nutrient concentration and are subsequently used to predict growth and interaction dynamics under different initial nutrient concentrations [7, 19]. Although some models were capable of predicting microbial growth under a limited range of initial nutrient concentrations [10], they also showed that the kinetics parameters estimated under various other ambient conditions had to be adjusted to provide an optimal fit under all considered conditions [10]. Unsurprisingly, studies have reported sensitivity of the model outcomes to small changes in the parameters associated with the nutrient uptake rate [12].

Our approach differs from previous ones as it considers nutrient uptake and growth parameters as a function of the initial nutrient concentration in the environment. In particular, the maximal nutrient uptake rate and biomass yield per unit of nutrient are both decreasing functions of initial nutrient concentration. While both empirical [34–37] and theoretical [10, 12, 32, 35, 45, 46] studies previously highlighted that the maximal nutrient uptake rate should not be considered independent of nutrient conditions, to our knowledge we are the first to put forward a functional form of this relationship as described in (8). We observe this relationship for a range of different microbial species including human fungal pathogens *C. albicans* (Fig 1(a), top panel), *C. glabrata* (Fig 1(b), top panel), baker’s yeast *S. cerevisiae* (Fig 1(c), top panel), and common coliform bacteria *E. coli* (Fig 1(d), top panel). This relationship was not sensitive to media enrichment or acidification (see S1 Text, Appendix C for details) nor to the addition of the lag-phase growth term into the model (2) (as described in S1 Text, Appendix D). Moreover, it was also evident on resources that are metabolised differently, both glucose that is directly imported into cells (Fig 1(a), 1(b) and 1(d)) and sucrose that is extracellularly hydrolysed before the uptake of its products [59] (Fig 1(c)).

Past studies proposed a qualitatively similar relationship between the maximal uptake rate and the initial nutrient concentration to the one we derive in Eq (8) [32, 35, 45, 46]. While some of these studies [32, 35] predicted a similar magnitude of the maximal uptake rate change across two orders of magnitude of nutrient concentrations as we see here, none have specified a functional form.

But what is the mechanistic basis behind this seemingly widely observed negative relationship between the maximal uptake rate and the initial nutrient concentration? In [32] the authors reasoned that the maximal uptake rate is proportional to the number of uptake sites expressed by the cell. They argued that, at low initial nutrient concentrations the cell is starved of nutrients and therefore it upregulates the expression of uptake sites, which is, however, limited by the cell’s size. In contrast, when the ambient nutrient is in excess the cell downregulates the expression of uptake sites while maintaining sufficient nutrient uptake.

Another explanation could be that nutrient uptake kinetics can change in response to nutrient conditions through various additional mechanisms. Microbes express a variety of transporter proteins that have differing nutrient uptake kinetics, and individual transporters can be removed, inactivated or have their affinities modified [42]. Such changes have been experimentally shown to result in an increase in the maximal rate of uptake at low resources [60], as is captured by the model proposed here. Both of these accounts could explain why micro-organisms might follow a different short-term steady-state uptake strategy at each initial nutrient concentration.

Finally, it has been shown in [32] that with decreasing concentration of ambient nutrient the difference in predicted growth between a constant maximal uptake rate and a non-constant maximal uptake rate becomes more apparent. Our results are consistent with this assertion indicating a significant difference between growth predictions based on our approach (taking into account that the maximal uptake rate is not constant) and predictions using fixed growth parameters (assuming that the maximal uptake rate is constant) at low initial nutrient concentrations (see Figs 3, 4 and 6(a)).

Our model also takes into account a decreasing relationship between initial nutrient concentration and cell yield. A functional form of this relationship has recently been derived in [33] from the first principles of nutrient uptake and metabolic processes in a cell. Such a relationship can be seen in our data for *C. albicans* (Fig 1(a), bottom panel), *C. glabrata* (Fig 1(b), bottom panel), *S. cerevisiae* (Fig 1(c), bottom panel), and *E. coli* (Fig 1(d), bottom panel) and was not found to be sensitive to media enrichment or acidification (see S1 Text, Appendix C for details) nor to the addition of the lag-phase growth term into the model (2) (as described in S1 Text, Appendix D).

We show that our approach performs significantly better at predicting microbial growth and the outcomes of between-species competition across different initial nutrient concentrations (Figs 3, 4 and 6(a)), compared to assuming fixed growth and uptake kinetic parameters. We utilise the two relationships: R1) between the maximal nutrient uptake rate and the initial nutrient concentration; and R2) between biomass yield and the initial nutrient concentration derived from empirical data on microbial growth on three nutrient concentrations within an interval of interest (Step 1 in Examples 1 and 2). Although having a larger amount of empirical data to describe the functional form of these two relationships would clearly contribute to understanding and characterising a given microbe’s growth, we demonstrate that the minimal requirement of three initial nutrient concentrations, chosen reasonably across the nutrient range of interest, is sufficient to significantly improve predictions compared to the fixed parameter approach.

Considering the maximal nutrient uptake rate and the biomass yield as functions of the initial nutrient concentrations, as we did here, is one of the improvements that can be made to the classical models of microbial growth dynamics. However, other factors shaping nutrient uptake kinetics missing from the classical ecological models including ours can also play an important role in predicting microbial growth. For example, the uptake kinetics parameters could vary during batch culture growth, with higher parameter values during exponential growth than during stationary phase, as demonstrated by an example of *C. albicans* growing on glucose [61]. Furthermore, the same study shows that even during stationary phase *C. albicans* can use two distinct uptake systems for glucose assimilation from the environment, each of which is characterised by different uptake kinetics parameters. Similar conclusions have been made for *E. coli* and *S. cerevisiae*, where different glucose uptake systems with dissimilar kinetics properties were found [40, 62]. Finally, a positive correlation between the nutrient uptake kinetics parameters and the cell size has been observed experimentally in different phytoplankton groups growing on phosphorus [35].

In general, a theoretical approach that allows the parameters describing nutrient uptake and growth kinetics to vary along different environmental conditions, can be deployed to predict microbial growth at different scales of organisation. In particular, the dynamic kinetic growth parameters described in our paper can readily be incorporated into ecological, population level models [7, 8], as well as metabolic genome-scale models that contain a Michaelis-Menten growth-modelling step [10, 12, 19]. However, due to its simplicity our ecological model has a certain advantage over genome-scale metabolic models; namely, we treat the enzyme kinetics as a black box and thus we do not require knowledge of detailed metabolic properties of a particular micro-organism to successfully predict its growth. In contrast, genome-scale models have complex parameterisation needs and require detailed metabolic reconstructions which are either challenging or not currently available for many non-laboratory cultured micro-organisms such as phytoplankton [63], deadly human pathogens [64] and plant pathogens [65].

However, our model has certain limitations, for example, it cannot accurately capture the lag phase (e.g. S1 Text, Appendix B, B4 Fig (a)) or features of the diauxic (biphasic) growth (e.g. S1 Text, Appendix B, B2 Fig (b)). But importantly, this can easily be rectified by building complexity into the model (2) as illustrated in S1 Text, Appendix D where we observed no departure from our key conclusions. Although our growth experiments were conducted in batch cultures, where waste products may accumulate and oxygen or nutrients other than the carbon source may become limiting, multiple factors demonstrate that our results are not exclusive to these conditions. Firstly, the trends we uncovered (Fig 1) are robust against media acidification and other nutrient enrichment (see S1 Text, Appendix C, C1 Fig). Secondly, we used protocols to enhance media shaking and gas exchange, while using organisms that can grow anaerobically (see section Growth experiments). Thirdly, previous studies have shown that the growth features which we find are not exclusive to microplate batch cultures. For instance, declining growth yields in increasing resource concentrations has been demonstrated in aerobic chemostat cultures [44], so are not the result of increasing oxygen deprivation. Moreover, such yield declines can arise by shifts in metabolic pathways, for example from fermentation to respiration, which can be triggered when sugar flux into the cell decreases [43, 44]. This switch in metabolism is irrespective of oxygen supply, which further suggests that when diauxic growth is observed in our study, it is a result of the rate of sugar uptake declining rather than oxygen deprivation. When additional intra-community interactions other than competition for the uptake of nutrients occur, such as e.g. by the production of public goods [59] or anti-competitor toxins [66], our model can easily be adapted and extended to consider more complex interactions among species in competition in order to predict community outcomes. Our approach which uses variable growth kinetic parameters can thus be considered as a foundation for various model scenarios, providing a common ground for the reliable estimation of microbial growth parameters.

## Supporting information

S1 Text Appendix A—Supplementary tables; Appendix B—Supplementary figures; Appendix C—Media acidification and alternative limiting nutrients; Appendix D—An extended mathematical model with a lag term. (PDF)
