# Supplementary material for: Predicting microbial growth dynamics in response to nutrient availability
Source: PLoS Comput Biol. 2021 Mar 18;17(3):e1008817. doi: 10.1371/journal.pcbi.1008817 (PMC8009381; doi:10.1371/journal.pcbi.1008817)
Supplement: S1 Text — (PDF) [file pcbi.1008817.s001.pdf]

Supplementary Information for:

Predicting microbial growth dynamics in response to nutrient availability

Olga A. Nev[1], Richard J. Lindsay[1◑], Alys Jepson[1◑], Lisa Butt[1], Robert E. Beardmore[1], Ivana Gudelj[1*]

1 Biosciences and Living Systems Institute, University of Exeter, Exeter, UK

◑These authors contributed equally to this work

* i.gudelj@exeter.ac.uk

Appendix A. Supplementary Tables. The optimal estimates for the parameters Y, V, and K (A1 Table – A7 Table, A10 Table – A13 Table) are obtained by means of the non-linear least-squares fit of the model (2) from the main text to the experimental data using *Mathematica*'s build in tools with a method *Gradient*. We observed that the reliability of the estimated parameters V, Y, K as well as the quality of the fit of the model (2) from the main text to the experimental data do not depend on the initial resource concentration (see SE for the fitted parameters in different resource concentrations in A1 Table – A7 Table, A10 Table – A13 Table and 95% confidence bands for the model fit shown in B1 Fig – B7 Fig).

A8 Table and A9 Table contain RMSE values calculated for different approaches for predicting growth described in the main text under all considered environmental conditions by means of the following formula:

$$\text{RMSE} = \sqrt{\frac{\sum_{i=1}^{n}(x_{\text{f},i} - x_{\text{o},i})^2}{n}}\,, \qquad (1)$$

where $x_{\text{o},i}$ is an experimentally observed value i, whereas $x_{\text{f},i}$ is a value predicted by function f for the experimental observation i, thus the term $(x_{\text{f},i} - x_{\text{o},i})$ represents a residual corresponding to the experimental observation i, and n is the total number of observations. The RMSE is a measurement of how concentrated the experimental data is around a predictive curve, so quantitatively estimates which prediction better describes the data (the lower the RMSE value, the better the prediction).

A1 Table. Optimal parameter values obtained by fitting model (2) from the main text to the experimental data on *C. albicans* growth on glucose (see B1 Fig).

	Initial glucose concentration (%)	Fitted V value (pmol of glucose/cell/min)	SE	Fitted K value (pmol of glucose/ml)	SE	Fitted Y value (cells/pmol of glucose)	SE
(a)	0.1	15.81	0.61	10^{11}	9.64×10^{-11}	10.6	0.09
(b)	0.3	7.05	0.16	10^{11}	1.09×10^{-11}	8.66	0.06
(c)	0.5	5.24	0.09	10^{11}	4.61×10^{-12}	6.56	0.04
(d)	1	3.89	0.03	10^{11}	1.21×10^{-12}	3.87	0.01
(e)	1.4	3.67	0.03	10^{11}	9.4×10^{-13}	2.85	0.01

A2 Table. Optimal parameter values obtained by fitting model (2) from the main text to the experimental data on *C. glabrata* growth on glucose (see B2 Fig).

	Initial glucose concentration (%)	Fitted V value (pmol of glucose/cell/min)	SE	Fitted K value (pmol of glucose/ml)	SE	Fitted Y value (cells/pmol of glucose)	SE
(a)	0.1	0.97	0.02	10^{10}	2.38×10^{-12}	17.36	0.13
(b)	0.3	0.3	0.004	10^{10}	1.06×10^{-13}	15.81	0.08
(c)	1.4	0.17	0.0005	10^{10}	7.49×10^{-15}	7.31	0.01
(d)	2	0.12	0.0005	10^{10}	5.33×10^{-15}	5.21	0.006
(e)	4	0.11	0.0009	10^{10}	9.02×10^{-15}	2.85	0.008

A3 Table. Optimal parameter values obtained by fitting model (2) from the main text to the experimental data on *S. cerevisiae* growth on sucrose (see B3 Fig).

	Initial sucrose concentration (%)	Fitted V value (pmol of sucrose/cell/min)	SE	Fitted K value (pmol of sucrose/ml)	SE	Fitted Y value (cells/pmol of sucrose)	SE
(a)	0.0625	14.57	0.11	10^{11}	1.63×10^{-11}	19.14	0.04
(b)	0.25	6.55	0.03	10^{11}	2.18×10^{-12}	12.05	0.02
(c)	1	5.28	0.03	10^{11}	1.35×10^{-12}	4.46	0.01
(d)	4	4.43	0.01	10^{11}	5.31×10^{-13}	1.18	0.001

A4 Table. Optimal parameter values obtained by fitting model (2) from the main text to the experimental data on *E. coli* growth on glucose (see B4 Fig).

	Initial glucose concentration (%)	Fitted V value (pmol of glucose/cell/min)	SE	Fitted K value (pmol of glucose/ml)	SE	Fitted Y value (cells/pmol of glucose)	SE
(a)	0.05	1.57	0.04	10^{11}	6.35×10^{-13}	140.26	1.67
(b)	0.1	1.4	0.02	10^{11}	3.03×10^{-13}	99.36	0.79
(c)	0.2	0.94	0.01	10^{11}	1.31×10^{-13}	67.76	0.55
(d)	0.4	0.85	0.01	10^{11}	7.44×10^{-14}	37.9	0.22

A5 Table. Optimal parameter values obtained by fitting model (2) from the main text to the experimental data on *C. glabrata* growth on glucose. For each of the initial glucose concentrations $N_{0,1} = 0.025\%$, $N_{0,2} = 1\%$, and $N_{0,3} = 2\%$, we list optimal parameter values V_i, K_i, and Y_i ($i = 1..3$) obtained by fitting the model (2) from the main text to the experimental data on *C. glabrata* growth on glucose (see B5 Fig). Note that we refer to V_i, K_i, and Y_i in the context of Example 1 from the main text, while the same parameters were labelled V_{G}^i, K_{G}^i, and Y_{G}^i in Example 2 from the main text, with the subscript G distinguishing *C. glabrata* data used to parameterise the model of competition with *C. albicans*.

	Initial glucose concentration (%)	Fitted value V_i (pmol of glucose/cell/min)	SE	Fitted value K_i (pmol of glucose/ml)	SE	Fitted value Y_i (cells/pmol of glucose)	SE
(1)	0.025	2.35	0.14	10^{10}	3.21×10^{-11}	19.32	0.26
(2)	1	0.19	0.001	10^{10}	1.6×10^{-14}	8.96	0.03
(3)	2	0.12	0.002	10^{10}	1.14×10^{-14}	5.36	0.05

A6 Table. Optimal parameter values obtained by fitting model (2) from the main text to the experimental data on *C. glabrata* growth on glucose. For each of the initial glucose concentrations $N_{0,1} = 0.2\%$, $N_{0,2} = 1\%$, and $N_{0,3} = 1.6\%$, we list optimal parameter values V_i, K_i, and Y_i ($i = 1..3$) obtained by fitting the model (2) from the main text to the experimental data on *C. glabrata* growth on glucose (see B6 Fig).

	Initial glucose concentration (%)	Fitted value V_i (pmol of glucose/cell/min)	SE	Fitted value K_i (pmol of glucose/ml)	SE	Fitted value Y_i (cells/pmol of glucose)	SE
(1)	0.2	0.51	0.009	10^{10}	4.34×10^{-13}	17	0.12
(2)	1	0.19	0.001	10^{10}	1.6×10^{-14}	8.96	0.03
(3)	1.6	0.14	0.001	10^{10}	9.97×10^{-15}	6.3	0.03

A7 Table. Optimal parameter values obtained by fitting model (2) from the main text to the experimental data on *C. albicans* growth on glucose. For each of the initial glucose concentrations $N_{0,1} = 0.025\%$, $N_{0,2} = 0.5\%$, and $N_{0,3} = 1\%$, we list optimal parameter values V_{A}^i, K_{A}^i, and Y_{A}^i ($i = 1..3$) obtained by fitting the model (2) from the main text to the experimental data on *C. albicans* growth on glucose (see B7 Fig).

	Initial glucose concentration (%)	Fitted value V_{A}^i (pmol of glucose/cell/min)	SE	Fitted value K_{A}^i (pmol of glucose/ml)	SE	Fitted value Y_{A}^i (cells/pmol of glucose)	SE
(1)	0.025	28.46	2.69	10^{11}	7.67×10^{-10}	16.94	0.34
(2)	0.5	4.77	0.13	10^{11}	6.25×10^{-12}	6.54	0.07
(3)	1	3.62	0.03	10^{11}	1.18×10^{-12}	3.8	0.02

A8 Table. RMSE values calculated for two approaches used to predict microbial growth inside the interval of initial nutrient concentrations. Since the RMSE values for the approach based on variable growth parameters are less than the RMSE values for the approach based on fixed growth parameters, we can conclude that our approach performs better.

Initial nutrient concentration (%)	Approach based on fixed growth parameters	Approach based on variable growth parameters
0.1	7.1×10^7	6.5×10^6
0.4	1.6×10^8	3.3×10^7
1.2	1.1×10^8	1.3×10^7
1.8	4.1×10^8	4.9×10^7

A9 Table. RMSE values calculated for two approaches used to predict microbial growth outside the interval of initial nutrient concentrations. Since the RMSE values for the approach based on variable growth parameters are less than the RMSE values for the approach based on fixed growth parameters, we can conclude that our approach performs better.

Initial nutrient concentration (%)	Approach based on fixed growth parameters	Approach based on variable growth parameters
0.1	7.1×10^7	1.6×10^7
1.8	4.1×10^8	3.8×10^7

A10 Table. Optimal parameter values obtained by fitting model (2) from the main text to the experimental data on *C. glabrata* growth on glucose in the control medium (see C1 Fig (a)).

	Initial glucose concentration (%)	Fitted V value (pmol of glucose/cell/min)	SE	Fitted K value (pmol of glucose/ml)	SE	Fitted Y value (cells/pmol of glucose)	SE
(a)	0.1	1.04	0.01	10^{10}	1×10^{-12}	14.97	0.09
(b)	0.3	0.47	0.004	10^{10}	2×10^{-13}	10.94	0.05
(c)	1.4	0.16	0.001	10^{10}	8×10^{-15}	6.26	0.03
(d)	2	0.13	0.0004	10^{10}	4×10^{-15}	5.04	0.01
(e)	4	0.11	0.0006	10^{10}	6×10^{-15}	2.64	0.01

A11 Table. Optimal parameter values obtained by fitting model (2) from the main text to the experimental data on *C. glabrata* growth on glucose in the nutrient enriched medium (see C1 Fig (b)).

	Initial glucose concentration (%)	Fitted V value (pmol of glucose/cell/min)	SE	Fitted K value (pmol of glucose/ml)	SE	Fitted Y value (cells/pmol of glucose)	SE
(a)	0.1	0.72	0.004	10^{10}	3×10^{-13}	18.43	0.05
(b)	0.3	0.48	0.002	10^{10}	9×10^{-14}	11.5	0.03
(c)	1.4	0.18	0.001	10^{10}	1×10^{-14}	5.57	0.03
(d)	2	0.13	0.0005	10^{10}	5×10^{-15}	4.84	0.01
(e)	4	0.1	0.0006	10^{10}	5×10^{-15}	2.94	0.01

A12 Table. Optimal parameter values obtained by fitting model (2) from the main text to the experimental data on *C. glabrata* growth on glucose in the pH buffered medium (see C1 Fig (c)).

	Initial glucose concentration (%)	Fitted V value (pmol of glucose/cell/min)	SE	Fitted K value (pmol of glucose/ml)	SE	Fitted Y value (cells/pmol of glucose)	SE
(a)	0.1	0.81	0.005	10^{10}	4×10^{-13}	18.52	0.05
(b)	0.3	0.48	0.004	10^{10}	2×10^{-13}	11.08	0.05
(c)	1.4	0.21	0.002	10^{10}	5×10^{-14}	5.29	0.04
(d)	2	0.12	0.0003	10^{10}	3×10^{-15}	5.98	0.01
(e)	4	0.09	0.0003	10^{10}	2×10^{-15}	3.65	0.01

A13 Table. Optimal parameter values obtained by fitting model (2) to the experimental data on *E. coli* growth on glucose (see D1 Fig (b)).

	Initial glucose concentration (%)	Fitted V value (pmol of glucose/ cell/min)	SE	Fitted K value (pmol of glucose/ ml)	SE	Fitted Y value (cells/ pmol of glucose)	SE	Fitted l value (min)	SE
(a)	0.05	3.41	0.2	10^{11}	1×10^{-11}	202	17.7	267	41.9
(b)	0.1	2.5	0.16	10^{11}	2×10^{-11}	163	19	381	58.7
(c)	0.2	1.92	0.04	10^{11}	3×10^{-11}	140	8	531	30.5
(d)	0.4	1.42	0.02	10^{11}	1×10^{-11}	53.5	1.1	344	12.2

Appendix B. Supplementary Figures.

B1 Fig.

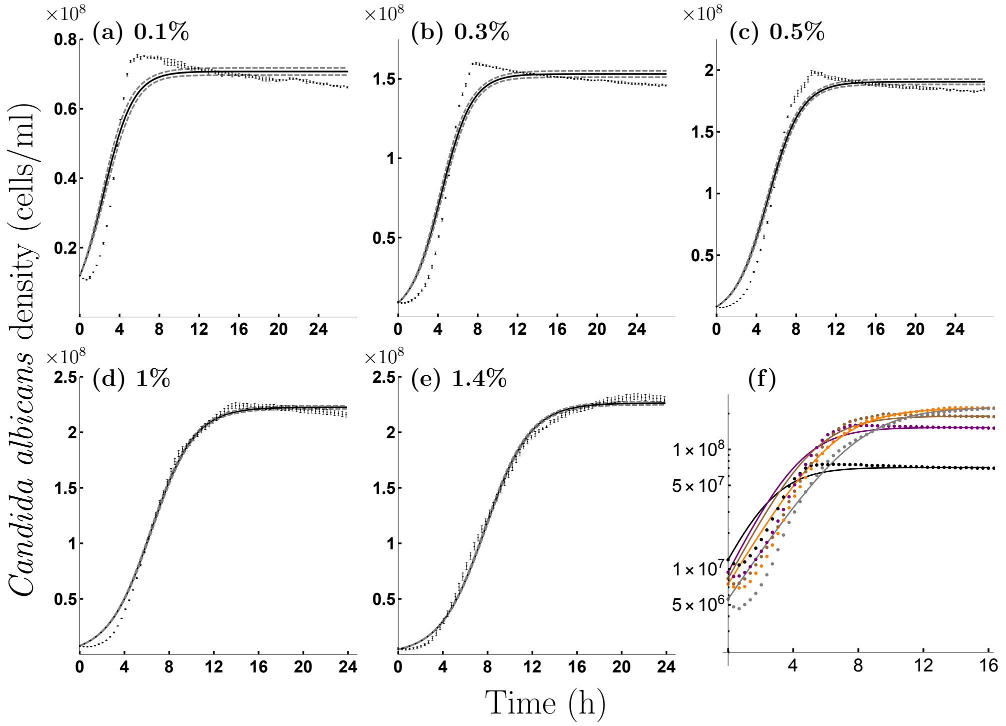

Growth of *C. albicans* on glucose. Experimental data on *C. albicans* growth in SC media with glucose together with the optimal fit of the model (2) from the main text for the following initial glucose concentrations: (a) 0.1%, (b) 0.3%, (c) 0.5%, (d) 1%, (e) 1.4%. Dots with error bars (mean values ± SE, where SE might be obscured by mean values, $n = 2$) represent experimental growth data, a solid line is the optimal non-linear least-squares fit of the model (2) from the main text to the data, and dashed lines show 95% confidence bands for the model fit. Estimated parameters values are as follows: (a) $V = 15.81$ pmol of glucose/cell/min, $K = 10^{11}$ pmol of glucose/ml, $Y = 10.6$ cells/pmol of glucose; (b) $V = 7.05$ pmol of glucose/cell/min, $K = 10^{11}$ pmol of glucose/ml, $Y = 8.66$ cells/pmol of glucose; (c) $V = 5.24$ pmol of glucose/cell/min, $K = 10^{11}$ pmol of glucose/ml, $Y = 6.56$ cells/pmol of glucose; (d) $V = 3.89$ pmol of glucose/cell/min, $K = 10^{11}$ pmol of glucose/ml, $Y = 3.87$ cells/pmol of glucose; (e) $V = 3.67$ pmol of glucose/cell/min, $K = 10^{11}$ pmol of glucose/ml, $Y = 2.85$ cells/pmol of glucose (see A1 Table). (f) Experimental data on *C. albicans* growth in SC media with glucose together with the optimal fit of the model (2) from the main text as in (a) – (e) plotted on a

single semi-logarithmic plot using the following corresponding colors: (a) black; (b) purple; (c) brown; (d) orange; (e) gray.

B2 Fig.

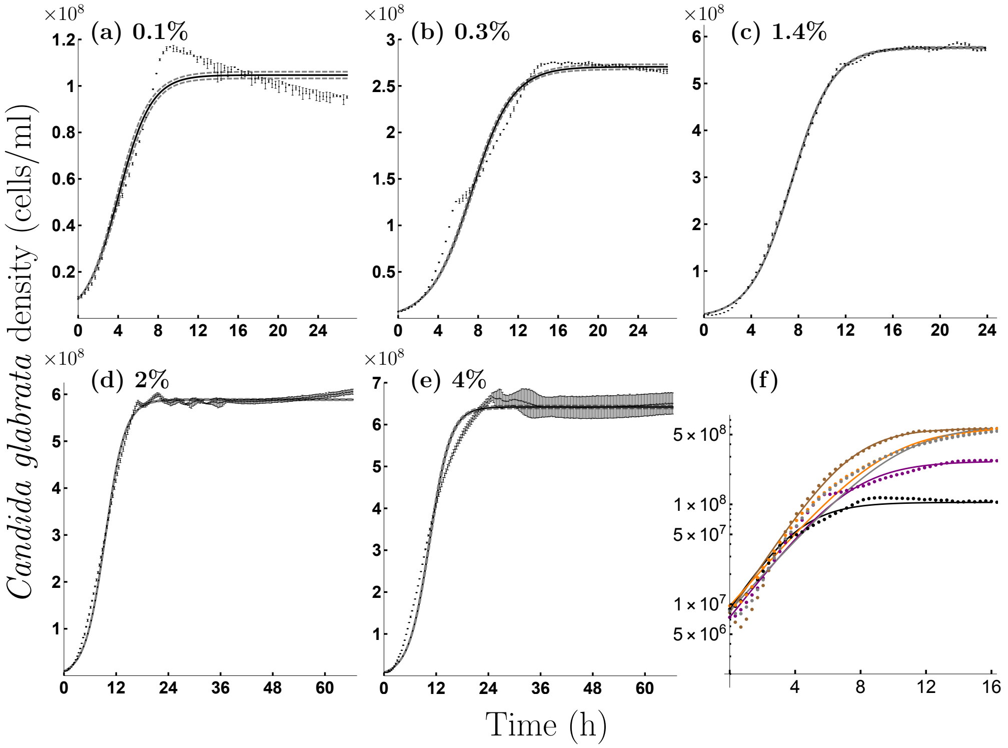

Growth of *C. glabrata* on glucose. Experimental data on *C. glabrata* growth in SC media with glucose together with the optimal fit of the model (2) from the main text for the following initial glucose concentrations: (a) 0.1%, (b) 0.3%, (c) 1.4%, (d) 2%, (e) 4%. Dots with error bars (mean values ± SE, where SE might be obscured by mean values, $n = 2$) represent experimental growth data, a solid line is the optimal non-linear least-squares fit of the model (2) from the main text to the data, and dashed lines show 95% confidence bands for the model fit. Estimated parameters values are as follows: (a) $V = 0.97$ pmol of glucose/cell/min, $K = 10^{10}$ pmol of glucose/ml, $Y = 17.36$ cells/pmol of glucose; (b) $V = 0.3$ pmol of glucose/cell/min, $K = 10^{10}$ pmol of glucose/ml, $Y = 15.81$ cells/pmol of glucose; (c) $V = 0.17$ pmol of glucose/cell/min, $K = 10^{10}$ pmol of glucose/ml, $Y = 7.31$ cells/pmol of glucose; (d) $V = 0.12$ pmol of glucose/cell/min, $K = 10^{10}$ pmol of glucose/ml, $Y = 5.21$ cells/pmol of glucose; (e) $V = 0.11$ pmol of glucose/cell/min, $K = 10^{10}$ pmol of glucose/ml, $Y = 2.85$ cells/pmol of glucose (see A2 Table). (f) Experimental data on *C. glabrata* growth in SC media with glucose together

with the optimal fit of the model (2) from the main text as in (a) – (e) plotted on a single semi-logarithmic plot using the following corresponding colors: (a) black; (b) purple; (c) brown; (d) orange; (e) gray.

B3 Fig.

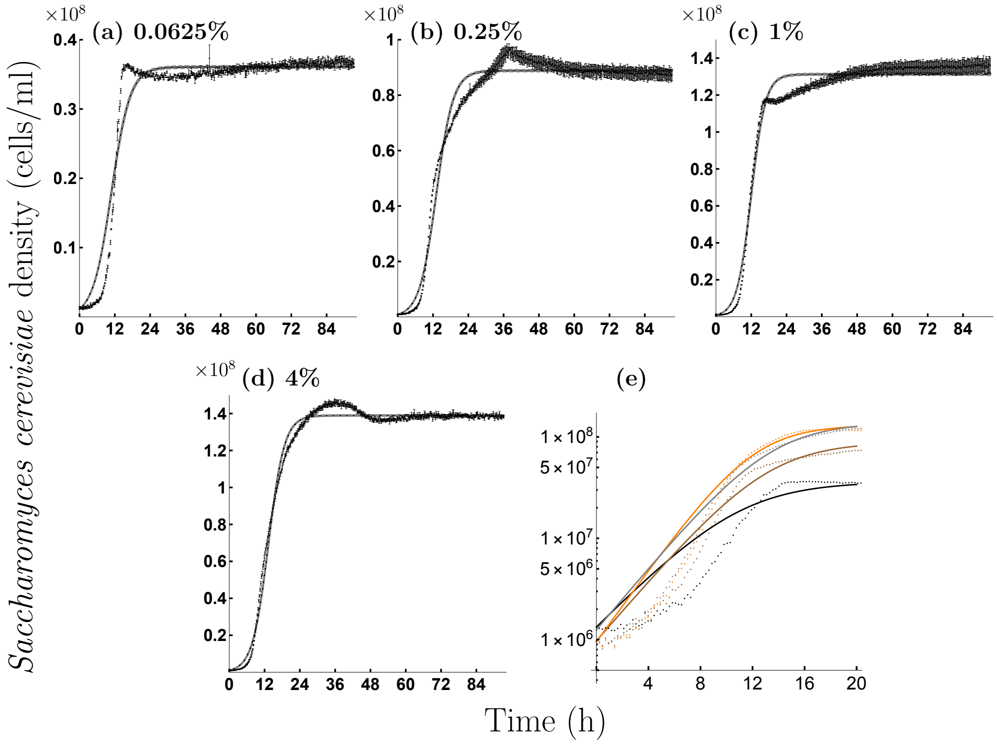

Growth of *S. cerevisiae* in sucrose. Experimental data on *S. cerevisiae* growth in SC media with sucrose (previously published by us in Fig. S2 of [1]) together with the optimal fit of the model (2) from the main text for the following initial sucrose concentrations: (a) 0.0625%, (b) 0.25%, (c) 1%, (d) 4%. Dots with error bars (mean values ± SE, where SE might be obscured by mean values, $n = 3$) represent experimental growth data, a solid line is the optimal non-linear least-squares fit of the model (2) from the main text to the data, and dashed lines show 95% confidence bands for the model fit. Estimated parameters values are as follows: (a) $V = 14.57$ pmol of sucrose/cell/min, $K = 10^{11}$ pmol of sucrose/ml, $Y = 19.14$ cells/pmol of sucrose; (b) $V = 6.55$ pmol of sucrose/cell/min, $K = 10^{11}$ pmol of sucrose/ml, $Y = 12.05$ cells/pmol of sucrose; (c) $V = 5.28$ pmol of sucrose/cell/min, $K = 10^{11}$ pmol of sucrose/ml, $Y = 4.46$ cells/pmol of sucrose; (d) $V = 4.43$ pmol of sucrose/cell/min, $K = 10^{11}$ pmol of sucrose/ml, $Y = 1.18$ cells/pmol of sucrose (see A3 Table). (e) Experimental data on

S. cerevisiae growth in SC media with sucrose together with the optimal fit of the model (2) from the main text as in (a) – (d) plotted on a single semi-logarithmic plot using the following corresponding colors: (a) black; (b) brown; (c) orange; (d) gray.

B4 Fig.

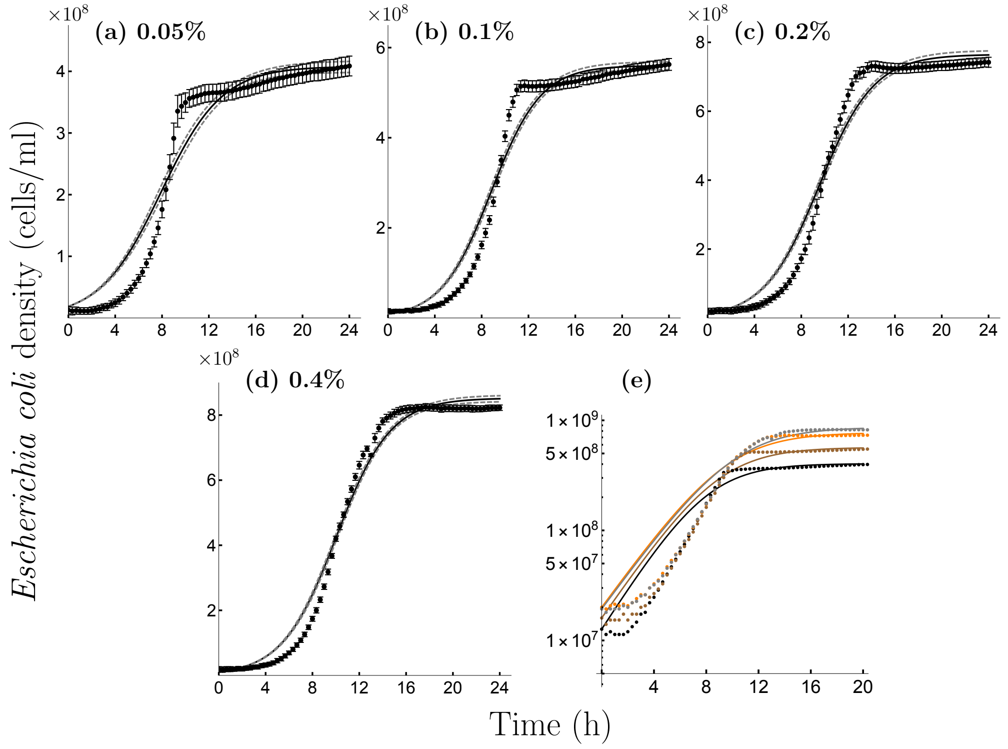

Growth of *E. coli* on glucose. Experimental data on *E. coli* growth in DM media with glucose together with the optimal fit of the model (2) from the main text for the following initial glucose concentrations: (a) 0.05%, (b) 0.1%, (c) 0.2%, (d) 0.4%. Dots with error bars (mean values ± SE, where SE might be obscured by mean values, $n = 3$) represent experimental growth data, a solid line is the optimal non-linear least-squares fit of the model (2) from the main text to the data, and dashed lines show 95% confidence bands for the model fit. Estimated parameters values are as follows: (a) $V = 1.57$ pmol of glucose/cell/min, $K = 10^{11}$ pmol of glucose/ml, $Y = 140.26$ cells/pmol of glucose; (b) $V = 1.4$ pmol of glucose/cell/min, $K = 10^{11}$ pmol of glucose/ml, $Y = 99.36$ cells/pmol of glucose; (c) $V = 0.94$ pmol of glucose/cell/min, $K = 10^{11}$ pmol of glucose/ml, $Y = 67.76$ cells/pmol of glucose; (d) $V = 0.85$ pmol of glucose/cell/min, $K = 10^{11}$ pmol of glucose/ml, $Y = 37.9$ cells/pmol of glucose (see A4 Table). (e) Experimental data on *E. coli* growth in DM media with glucose together

with the optimal fit of the model (2) from the main text as in (a) – (d) plotted on a single semi-logarithmic plot using the following corresponding colors: (a) black; (b) brown; (c) orange; (d) gray.

B5 Fig.

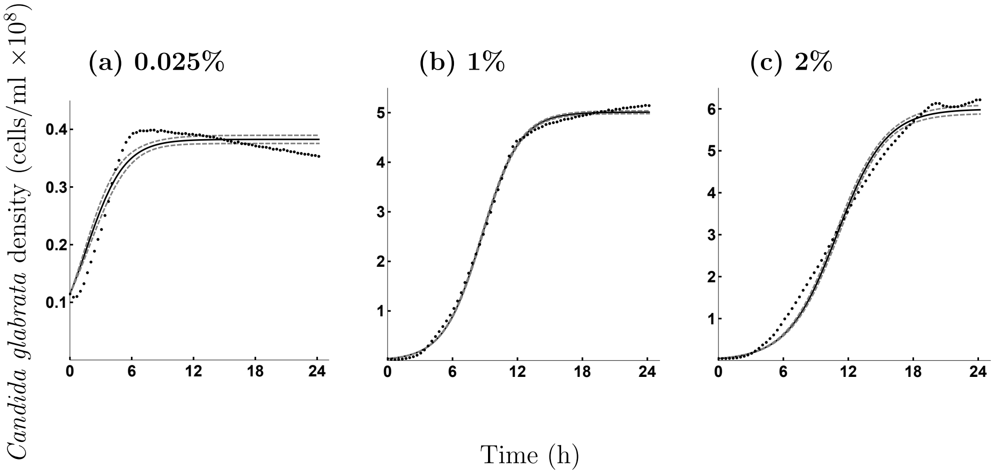

Growth of *C. glabrata* on glucose. Experimental data on *C. glabrata* growth in SC media with glucose together with the optimal fit of the model (2) from the main text for the following initial glucose concentrations: (a) 0.025%; (b) 1%; (c) 2%. Dots represent experimental growth data, a solid line is the optimal non-linear least-squares fit of the model (2) from the main text to the data, and dashed lines show 95% confidence bands for the model fit. Estimated parameters values are as follows: (a) $V = 2.35$ pmol of glucose/cell/min, $K = 10^{10}$ pmol of glucose/ml, $Y = 19.32$ cells/pmol of glucose; (b) $V = 0.19$ pmol of glucose/cell/min, $K = 10^{10}$ pmol of glucose/ml, $Y = 8.96$ cells/pmol of glucose; (c) $V = 0.12$ pmol of glucose/cell/min, $K = 10^{10}$ pmol of glucose/ml, $Y = 5.36$ cells/pmol of glucose (see A5 Table).

B6 Fig.

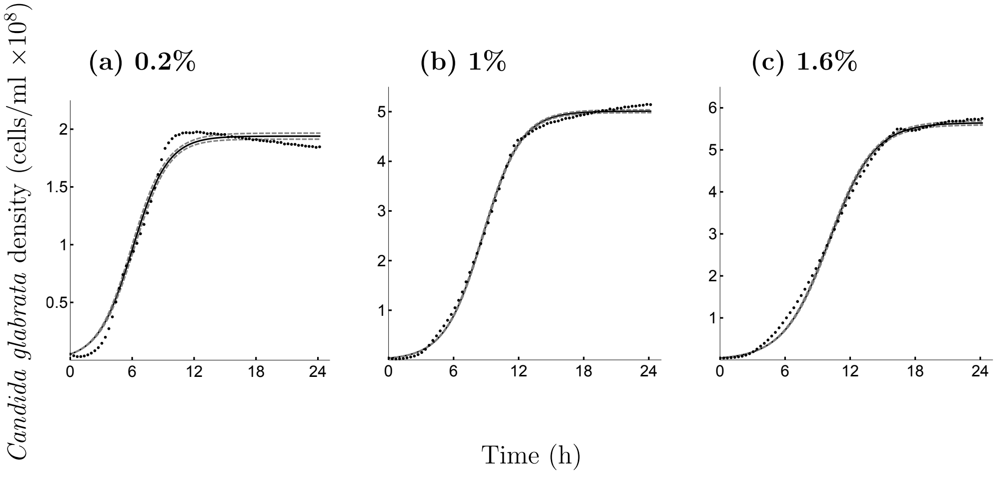

Growth of *C. glabrata* on glucose. Experimental data on *C. glabrata* growth in SC media with glucose together with the optimal fit of the model (2) from the main text for the following initial glucose concentrations: (1) 0.2%; (2) 1%; (3) 1.6%. Dots represent experimental growth data, a solid line is the optimal non-linear least-squares fit of the model (2) from the main text to the data, and dashed lines show 95% confidence bands for the model fit. Estimated parameters values are as follows: (1) $V = 0.51$ pmol of glucose/cell/min, $K = 10^{10}$ pmol of glucose/ml, $Y = 17$ cells/pmol of glucose; (2) $V = 0.19$ pmol of glucose/cell/min, $K = 10^{10}$ pmol of glucose/ml, $Y = 8.96$ cells/pmol of glucose; (3) $V = 0.14$ pmol of glucose/cell/min, $K = 10^{10}$ pmol of glucose/ml, $Y = 6.3$ cells/pmol of glucose (see A6 Table).

B7 Fig.

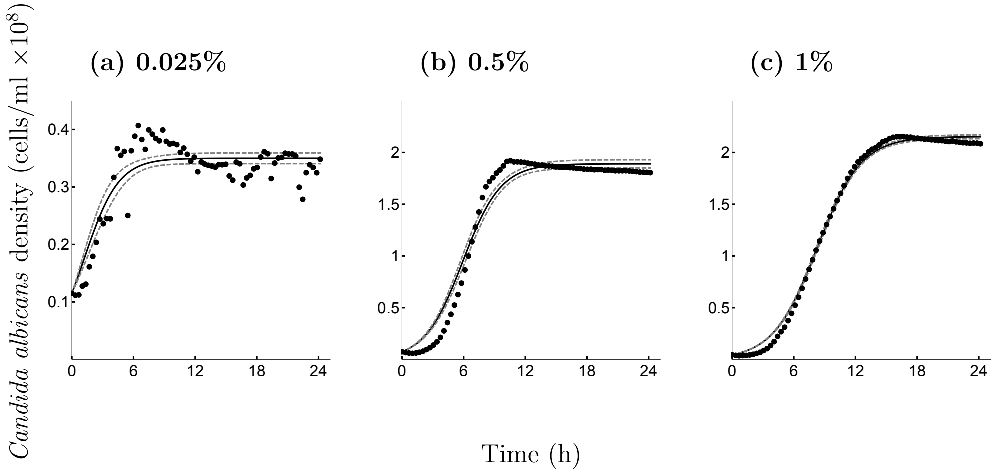

Growth of *C. albicans* on glucose. Experimental data on *C. albicans* growth in SC

media with glucose together with the optimal fit of the model (2) from the main text for the following initial glucose concentrations: (1) 0.025%; (2) 0.5%; (3) 1%. Dots represent experimental growth data, a solid line is the optimal non-linear least-squares fit of the model (2) from the main text to the data, and dashed lines show 95% confidence bands for the model fit. Estimated parameters values are as follows: (1) $V = 28.46$ pmol of glucose/cell/min, $K = 10^{11}$ pmol of glucose/ml, $Y = 16.94$ cells/pmol of glucose; (2) $V = 4.77$ pmol of glucose/cell/min, $K = 10^{11}$ pmol of glucose/ml, $Y = 6.54$ cells/pmol of glucose; (3) $V = 3.62$ pmol of glucose/cell/min, $K = 10^{11}$ pmol of glucose/ml, $Y = 3.8$ cells/pmol of glucose (see A7 Table).

B8 Fig.

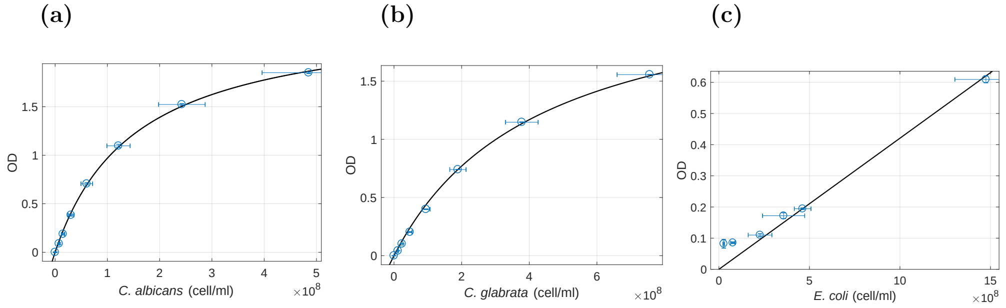

Conversion from optical density to CFU of (a) *C. albicans*, (b) *C. glabrata*, and (c) *E. coli* growing on glucose. (a) and (b): Serial dilutions of an initial culture were prepared in a 48-well plate and their OD measured. The number of cell/ml in the initial culture was calculated using a haemocytometer. The fitted curve uses the function cell/ml $= -OD \times c/(OD - OD_{\max})$, where OD is the optical density measured, and c and $OD_{\max}$ are fitted parameters. For *C. albicans* we found $OD_{\max} = 2.5 \pm 0.2$ and $c = (1.5 \pm 0.2) \times 10^8$ cell/ml. For *C. glabrata* we found $OD_{\max} = 2.4 \pm 0.2$ and $c = (4.3 \pm 0.8) \times 10^8$ cell/ml. (c): Serial dilutions were prepared in a 96-well plate and their OD measured. The CFU in each dilution was determined using platecounting. The fitted line gives cell/ml $= OD \times c$, with $c = (2.3 \pm 0.3) \times 10^9$ cell/ml.

Appendix C. Media acidification and alternative limiting nutrients. The growth assays used to estimate the relationship between the initial nutrient concentration and parameters V and Y (see Fig 1 in the main text) were conducted in batch cultures using SC media for *Candida spp.* and *S.cerevisiae* or DM media for *E.coli* (for details see Materials and methods section in the main text). Glucose was assumed to be the limiting nutrient in *Candida spp.* and *E.coli* experiments, while in *S.cerevisiae* experiments it was sucrose. Since in batch cultures nutrients are not replenished nor waste products removed, two additional factors, other than the limiting sugar, could affect microbial growth. First, the media can become acidified due to the secretion of organic acids [2] and second, individual nutrient components such as nitrogen, could become limiting.

To test whether the observed relationships between the initial nutrient concentration and parameters V and Y could result from either of these factors we repeated the growth assays for *C. glabrata* in control (C1 Fig (a)) and modified growth media (C1 Fig (b) and (c)). While the control media was the same as in B2 Fig, in addition we used enriched media at 1.8×the concentration of the control media as well as control media that was additionally buffered with 0.1M potassium phosphate (pH 6) (see Materials and methods section in the main text). We found that negative relationships between initial glucose concentration and parameters V and Y (see C1 Fig (d) and (e), respectively) were observed in control (black line), nutrient enriched (gray line), and pH buffered (brown line) media.

But is glucose the only limiting nutrient in our experiments? While it has previously been shown that yeast completely deplete 2% glucose in SC media [2], this might not be the case for 4% glucose. Indeed, minor increases in the estimated values of Y at 4% glucose were observed in nutrient enriched and pH buffered media compared to the control media (see C1 Fig (e)). This could suggest that nutrients, other than the carbon source, might be limiting growth in control media and so yield might be restricted by factors other than metabolic inefficiencies [3]. To rule this scenario out we carried out an experiment using an engineered strain of *S. cerevisiae* (TM6*) that has fully respiratory metabolism resulting in a much higher metabolic efficiency compared to the wild-type's (CEN.PK2-1C) respiro-fermentative metabolism [4]. We found that the TM6* strain was able to reach a substantially higher yield than the wild-type in 4%

sucrose media (see C1 Fig (f) and (g)). Thus we argue that the observed decline in yield from low to high glucose environments is the result of metabolic inefficiencies known to constrain microbial growth at high nutrient concentrations [5].

Collectively, these results indicate that the negative relationships between initial nutrient concentration and parameters V and Y (Fig 1 from the main text) are not sensitive to media acidification or enrichment.

C1 Fig.

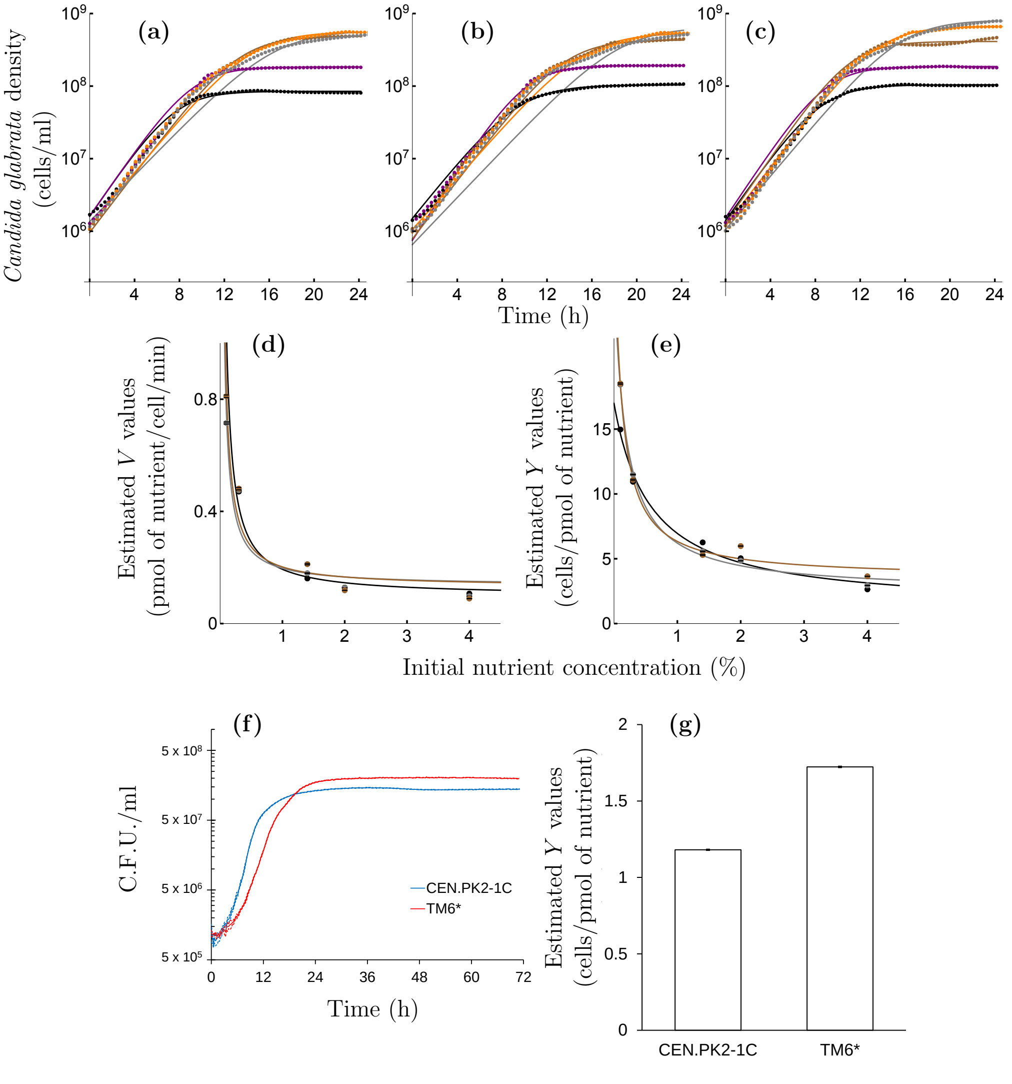

(a) Growth of *C. glabrata* on glucose in the control medium. Experimental data on *C. glabrata* growth in the SC medium with glucose together with the optimal fit of the model (2) from the main text plotted on a single semi-logarithmic plot for the following initial glucose concentrations: 0.1% (black), 0.3% (purple), 1.4% (brown), 2%

(orange), 4% (gray). Estimated parameters values are as follows: (0.1%) $V = 1.04$ pmol of glucose/cell/min, $K = 10^{10}$ pmol of glucose/ml, $Y = 14.97$ cells/pmol of glucose; (0.3%) $V = 0.47$ pmol of glucose/cell/min, $K = 10^{10}$ pmol of glucose/ml, $Y = 10.94$ cells/pmol of glucose; (1.4%) $V = 0.16$ pmol of glucose/cell/min, $K = 10^{10}$ pmol of glucose/ml, $Y = 6.26$ cells/pmol of glucose; (2%) $V = 0.13$ pmol of glucose/cell/min, $K = 10^{10}$ pmol of glucose/ml, $Y = 5.04$ cells/pmol of glucose; (4%) $V = 0.11$ pmol of glucose/cell/min, $K = 10^{10}$ pmol of glucose/ml, $Y = 2.64$ cells/pmol of glucose (see A10 Table).

(b) Growth of *C. glabrata* on glucose in the nutrient enriched medium. Experimental data on *C. glabrata* growth at 1.8×the concentration of the SC control medium with glucose together with the optimal fit of the model (2) from the main text plotted on a single semi-logarithmic plot for the following initial glucose concentrations: 0.1% (black), 0.3% (purple), 1.4% (brown), 2% (orange), 4% (gray). Estimated parameters values are as follows: (0.1%) $V = 0.72$ pmol of glucose/cell/min, $K = 10^{10}$ pmol of glucose/ml, $Y = 18.43$ cells/pmol of glucose; (0.3%) $V = 0.48$ pmol of glucose/cell/min, $K = 10^{10}$ pmol of glucose/ml, $Y = 11.5$ cells/pmol of glucose; (1.4%) $V = 0.18$ pmol of glucose/cell/min, $K = 10^{10}$ pmol of glucose/ml, $Y = 5.57$ cells/pmol of glucose; (2%) $V = 0.13$ pmol of glucose/cell/min, $K = 10^{10}$ pmol of glucose/ml, $Y = 4.84$ cells/pmol of glucose; (4%) $V = 0.1$ pmol of glucose/cell/min, $K = 10^{10}$ pmol of glucose/ml, $Y = 2.94$ cells/pmol of glucose (see A11 Table).

(c) Growth of *C. glabrata* on glucose in the pH buffered medium. Experimental data on *C. glabrata* growth in the SC control medium with glucose with the addition of 0.1M potassium phosphate together with the optimal fit of the model (2) from the main text plotted on a single semi-logarithmic plot for the following initial glucose concentrations: 0.1% (black), 0.3% (purple), 1.4% (brown), 2% (orange), 4% (gray). Estimated parameters values are as follows: (0.1%) $V = 0.81$ pmol of glucose/cell/min, $K = 10^{10}$ pmol of glucose/ml, $Y = 18.52$ cells/pmol of glucose; (0.3%) $V = 0.48$ pmol of glucose/cell/min, $K = 10^{10}$ pmol of glucose/ml, $Y = 11.08$ cells/pmol of glucose; (1.4%) $V = 0.21$ pmol of glucose/cell/min, $K = 10^{10}$ pmol of glucose/ml, $Y = 5.29$ cells/pmol of glucose; (2%) $V = 0.12$ pmol of glucose/cell/min, $K = 10^{10}$ pmol of glucose/ml, $Y = 5.98$ cells/pmol of glucose; (4%) $V = 0.09$ pmol of glucose/cell/min, $K = 10^{10}$ pmol of glucose/ml, $Y = 3.65$ cells/pmol of glucose (see A12 Table).

(d) Relationships between the maximal uptake rate parameter V and the initial nutrient concentration N_0 observed for *C. glabrata* growing on glucose. Dots with error bars (estimated values ± SE, where SE might be obscured by data points) represent optimal estimates for maximal uptake rate parameters V_i ($i = 1..5$) obtained by fitting the model (2) from the main text in the control medium (black), the nutrient enriched medium (gray), and the pH buffered medium (brown) to the experimental data on growth at the following initial glucose concentrations $N_{0,i}$ ($i = 1..5$): 0.1%, 0.3%, 1.4%, 2%, 4%, and a solid line is the optimal non-linear least-squares fit of the function (8) from the main text to the plotted data.
(e) Relationships between the yield parameter Y and the initial nutrient concentration N_0 observed for *C. glabrata* growing on glucose. Dots with error bars (estimated values ± SE, where SE might be obscured by data points) represent optimal estimates for yield parameters Y_i ($i = 1..5$) obtained by fitting the model (2) from the main text in the control medium (black), the nutrient enriched medium (gray), and the pH buffered medium (brown) to the experimental data on growth at the following initial glucose concentrations $N_{0,i}$ ($i = 1..5$): 0.1%, 0.3%, 1.4%, 2%, 4%, and a solid line is the optimal non-linear least-squares fit of the function (3) from the main text to the plotted data.
(f) Growth of *S. cerevisiae* in sucrose. Growth data for the engineered strain of *S. cereisiae* TM6* (red) and the wild-type strain CEN.PK2-1C (blue) in 4% sucrose SC media. The data is present as mean values (solid lines) ± SE (dashed lines, might be obscured by mean values, $n = 3$).
(g) Estimated values of the yield parameter Y for *S. cerevisiae* growing in sucrose. Bars with error bars (estimated values ± SE, where SE might be obscured by values) represent optimal estimates for the yield parameter Y obtained by fitting the model (2) from the main text to the experimental data on growth for the engineered strain of *S. cereisiae* TM6* (left, $Y = 1.72$ cells/pmol of sucrose) and the wild-type strain CEN.PK2-1C (right, $Y = 1.18$ cells/pmol of sucrose) in 4% sucrose media (see C1 Fig (f)).

Appendix D. An extended mathematical model with a lag term. Due to its simplicity, our model (2) from the main text cannot accurately capture the lag phase of microbial growth (e.g. as in the *E. coli*'s example, see B4 Fig). Here we extend the model (2) from the main text to include a lag-phase term as suggested by Baranyi et al. [6] to obtain:

$$\begin{cases} \dot{N}(t) = -q(N(t)) \times B(t) \\ \dot{B}(t) = Y \times q(N(t)) \times B(t) \times t^2/(l^2 + t^2) \end{cases} \qquad t \in [0, T] \;, \tag{2}$$

where $q(N(t))$ is as in Eq (1) from the main text, and l denotes the lag time. Numerical solutions of the extended model (2) were fitted to the experimental data on *E. coli* following the same procedure as before (see Appendix A), to estimate the parameters V, Y, K, and l. As in the case of the simple model (2) from the main text, we observed that the estimated values of V and Y decrease with increasing initial nutrient concentration, whereas the estimated value of K did not depend on the initial nutrient concentration. The results are presented in D1 Fig.

D1 Fig.

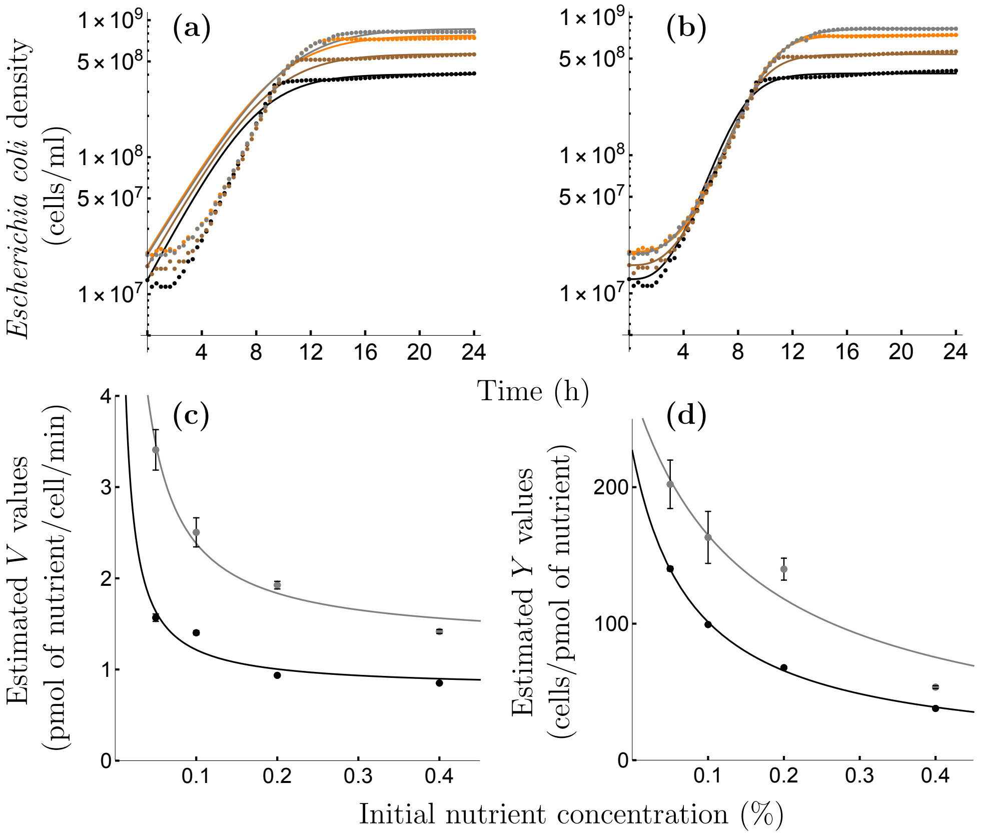

(a) Growth of *E. coli* on glucose. Experimental data on *E. coli* growth in DM media with glucose together with the optimal fit of the model (2) from the main text plotted on a single semi-logarithmic plot for the following initial glucose concentrations: 0.05% (black), 0.1% (brown), 0.2% (orange), 0.4% (gray). Estimated parameters values are as follows: (0.05%) $V = 1.57$ pmol of glucose/cell/min, $K = 10^{11}$ pmol of glucose/ml, $Y = 140.26$ cells/pmol of glucose; (0.1%) $V = 1.4$ pmol of glucose/cell/min, $K = 10^{11}$ pmol of glucose/ml, $Y = 99.36$ cells/pmol of glucose; (0.2%) $V = 0.94$ pmol of glucose/cell/min, $K = 10^{11}$ pmol of glucose/ml, $Y = 67.76$ cells/pmol of glucose; (0.4%) $V = 0.85$ pmol of glucose/cell/min, $K = 10^{11}$ pmol of glucose/ml, $Y = 37.9$ cells/pmol of glucose (see B4 Fig (e) and A4 Table).

(b) Growth of *E. coli* on glucose. Experimental data on *E. coli* growth in DM media with glucose together with the optimal fit of the model (2) plotted on a single semi-logarithmic plot for the following initial glucose concentrations: 0.05% (black), 0.1% (brown), 0.2% (orange), 0.4% (gray). Estimated parameters values are as follows:

(0.05%) $V = 3.41$ pmol of glucose/cell/min, $K = 10^{11}$ pmol of glucose/ml, $Y = 202$ cells/pmol of glucose, $l = 267$ min; (0.1%) $V = 2.5$ pmol of glucose/cell/min, $K = 10^{11}$ pmol of glucose/ml, $Y = 163$ cells/pmol of glucose, $l = 381$ min; (0.2%) $V = 1.92$ pmol of glucose/cell/min, $K = 10^{11}$ pmol of glucose/ml, $Y = 140$ cells/pmol of glucose, $l = 531$ min; (0.4%) $V = 1.42$ pmol of glucose/cell/min, $K = 10^{11}$ pmol of glucose/ml, $Y = 53.5$ cells/pmol of glucose, $l = 344$ min (see A13 Table).

(c) Relationships between the maximal uptake rate parameter V and the initial nutrient concentration N_0 observed for *E. coli* growing on glucose. Dots with error bars (estimated values ± SE, where SE might be obscured by values) represent optimal estimates for maximal uptake rate parameters V_i ($i = 1..4$) obtained by fitting the model (2) from the main text (black) and the model (2) (gray) to the experimental data on growth at the following initial glucose concentrations $N_{0,i}$ ($i = 1..4$): 0.05%, 0.1%, 0.2%, 0.4%, and a solid line is the optimal non-linear least-squares fit of the function (8) from the main text to the plotted data.

(d) Relationships between the yield parameter Y and the initial nutrient concentration N_0 observed for *E. coli* growing on glucose. Dots with error bars (estimated values ± SE, where SE might be obscured by values) represent optimal estimates for yield parameters Y_i ($i = 1..4$) obtained by fitting the model (2) from the main text (black) and the model (2) (gray) to the experimental data on growth at the following initial glucose concentrations $N_{0,i}$ ($i = 1..4$): 0.05%, 0.1%, 0.2%, 0.4%, and a solid line is the optimal non-linear least-squares fit of the function (3) from the main text to the plotted data.

References

1. Lindsay RJ, Pawlowska BJ, Gudelj I. Privatization of public goods can cause population decline. Nat Ecol Evol. 2019;3:1206–1216.

2. Burtner CR, Murakami CJ, Kennedy BK, Kaeberlein M. A molecular mechanism of chronological aging in yeast. Cell cycle. 2009;8:1256–1270.

3. Pfeiffer T, Schuster S, Bonhoeffer S. Cooperation and Competition in the Evolution of ATP-Producing Pathways. Science. 2001;292(5516):504–507.

4. Otterstedt K, Larsson C, Bill RM, Ståhlberg A, Boles E, Hohmann S, Gustafsson L. Switching the mode of metabolism in the yeast *Saccharomyces cerevisiae*. EMBO Rep. 2004;5:532–537.

5. Postma E, Verduyn C, Scheffers W. A., Van Dijken JP. Enzymic analysis of the crabtree effect in glucose-limited chemostat cultures of *Saccharomyces cerevisiae*. Appl Environ Microbiol. 1989;55:468–477.

6. Baranyi J, Roberts TA, McClure P. A non-autonomous differential equation to model bacterial growth. Food Microbiol. 1993;10:43–59.
